# Inflammation, Autoimmunity and Neurodegenerative Diseases, Therapeutics and Beyond

**DOI:** 10.2174/1570159X22666231017141636

**Published:** 2024-10-17

**Authors:** Jenny Valentina Garmendia, Claudia Valentina De Sanctis, Viswanath Das, Narendran Annadurai, Marián Hajduch, Juan Bautista De Sanctis

**Affiliations:** 1 Institute of Molecular and Translational Medicine, Faculty of Medicine and Dentistry, Palacky University, Olomouc, The Czech Republic;; 2 The Czech Advanced Technology and Research Institute (Catrin), Palacky University, Olomouc, The Czech Republic

**Keywords:** Neurodegenerative diseases (NDs), Alzheimer's disease (AD), Parkinson's disease (PD), β-amyloid, tau, α-synuclein, neurodegeneration, neuroinflammation, autoimmunity, therapy, vaccines

## Abstract

Neurodegenerative disease (ND) incidence has recently increased due to improved life expectancy. Alzheimer's (AD) or Parkinson's disease (PD) are the most prevalent NDs. Both diseases are poly genetic, multifactorial and heterogenous. Preventive medicine, a healthy diet, exercise, and controlling comorbidities may delay the onset. After the diseases are diagnosed, therapy is needed to slow progression. Recent studies show that local, peripheral and age-related inflammation accelerates NDs' onset and progression. Patients with autoimmune disorders like inflammatory bowel disease (IBD) could be at higher risk of developing AD or PD. However, no increase in ND incidence has been reported if the patients are adequately diagnosed and treated. Autoantibodies against abnormal tau, β amyloid and α- synuclein have been encountered in AD and PD and may be protective. This discovery led to the proposal of immune-based therapies for AD and PD involving monoclonal antibodies, immunization/vaccines, pro-inflammatory cytokine inhibition and anti-inflammatory cytokine addition. All the different approaches have been analysed here. Future perspectives on new therapeutic strategies for both disorders are concisely examined.

## 1 INTRODUCTION

### 1.1 General Overview

Neurodegeneration refers to the progressive deterioration and loss of function of neurons. Neurodegenerative diseases (ND) include Alzheimer's disease (AD), Amyotrophic lateral sclerosis (ALS), Friedreich ataxia, Frontotemporal lobular degeneration (FTLD), Huntington's disease (HD), Lewy body dementia, Multiple Sclerosis (MS), Parkinson's disease (PD), and Spinal muscular atrophy (SMA). All NDs have different origins. Genetic involvement in several NDs has been studied for years [1]. HD and ALS are primary examples. HD is a progressive brain disorder caused by a single defective gene on chromosome 4 that codifies for the protein huntingtin. The disease generally manifests between 30 and 50 years [2]. ALS is a neurodegenerative disease that affects motor neurons. Around 60% of ALS patients have a genetic correlation with the disease. The genes associated with ALS are: 1) superoxide dismutase 1 (SOD1), 2) TAR DNA‐binding protein (TDP-53), 3) fused in sarcoma (FUS) and 4) chromosome 9 open reading frame 72 (C9orf72) [3]. Some other genes have been recently discovered. MS is an autoimmune neurodegenerative disease associated with demyelination, inflammation and irreversible axonal loss [4]. Myelin is the target antigen, and demyelination results in axonal loss, the primary cause of irreversible neurological disability in MS [4-6]. MS's inflammatory aspects are unique compared to other NDs [4-6]. The immunogenetic analysis of MS patients has revealed a link between the polymorphisms of HLA [6]. HLA DRB1*15:01 and HLA DRB1*04 with younger age of onset, HLA DRB1*03 could be linked to pediatric forms, and MHLA-B*44:02 appears to be associated with less cortical atrophy and fewer MRI brain lesions [6]. Since AD and PD are the most common neurodegenerative disorders (4% of individuals over 65 and 1% of people over the age of 60, respectively), the review will focus mainly on these diseases [7, 8]. Genetic predisposition, malnutrition, the excessive use of legal and non-legal drugs, comorbidities and other environmental events may increase the prevalence and earlier onset of NDs.

## 2 GENETIC STUDIES IN AD AND PD

AD is characterised by 1) the presence of amyloid-β plaques, 2) the formation and deposition of neurofibrillary tangles (NFTs) composed of hyperphosphorylated tau proteins, 3) oxidative stress, 4) neuronal death starting from the hippocampus, 5) astrocytosis and microgliosis 6) neuroinflammation, and 7) NLRP3 inflammasome activation; all of which lead to progressive memory loss and eventually dementia [7, 9-10]. No simple relationship exists between specific genes and increased susceptibility to AD. It is a polygenic, multifactorial and heterogeneous disease. The early onset of the disease [11] has been linked to three main genes that are responsible for a particular form of pathology: amyloid precursor protein (APP) on chromosome 21, presenilin 1 (PSEN1) on chromosome 14, and presenilin 2 (PSEN2) on chromosome 1. Other genes involved in AD are ABCA1, ApoE3/4, CYP2D6, CHAT, CHRNA7, ADAMTS12, IL15, FHIT and ESR1. Many other pathology-related genes are linked to other neurodegenerative diseases, *i.e*. F5, MAP1B, and BCAS3 are related to AD and PD [12, 13]. Single nucleotide polymorphisms (SNP) of both pro- and antiinflammatory cytokines, IL-1, IL-6, TNFα, IL-4, IL-10, TGFβ [14], and its promoters, IL-1α -889, IL-6 -176, IL-8 -251, IL-10 -1082, IL-10 -819, and IL-18 -607 have been reported in AD [14, 15] and PD [15] suggesting a link with immune response. Recently, the upregulation of chemokines CCL5, CXCL1, and CXCL16 has been recorded in the brains of AD patients [16]; nonetheless, its role in leukocyte migration in CNS is still unclear. α-Synuclein is a presynaptic neuronal protein that appears to control neurotransmitter release. Mutations in the α-syn gene encoding, A18T, A29S, A30P, E46K, H50Q, G51D, A53E, and A53T are linked to familial PD [17]. The polymorphisms A18T, A29S, and A30P are associated with a typical PD phenotype and slight clinical characteristics; however, patients with polymorphisms E46K, H50Q, G51D, A53E, or A53T, manifest severe disease with rapid progression [17]. Polymorphisms in the human leukocyte antigen, HLA-DRA, HLA-DRB1 (rs660895) and HLA-DRB, IL-6 rs1800795, TNF-α rs1799964, PON1 rs854560, CYP2D6 rs3892097, BST1 rs11931532, and CCDC62 rs12817488 are also involved in PD [21-25]. They may be co-associated with other genetic markers of the disease [18-21]. Genetic screening may be helpful to families with one or more cases of these NDs. Preventive medicine, a healthy diet, exercise, and control of comorbidities may delay disease onset.

## 3 THE BLOOD-BRAIN BARRIER (BBB) AND THE BLOOD-SPINAL CORD BARRIER (BSCB)

The central nervous system (CNS) is highly regulated and guarded by physical barriers and specialised cells. The blood-brain barrier (BBB) and blood-spinal cord barrier (BSCB) are not physical barriers, but cells (pericytes) that heavily restrict the flow of molecules by tightening blood capillaries and the secreting VE-cadherin and P-glycoprotein [22]. These physical barriers can be more permeable with age [23]. The main difference between the BBB and the BSCB is the size of the zonula occludens (multiprotein complexes that prevent leakage of solutes and water between the epithelial cells) and the number of pericytes, allowing a more extensive range of molecules to pass through the barrier in BSCB. It is, therefore, assumed that the spinal cord may be more susceptible to inflammatory insults than the brain [24]. In neuroinflammatory disorders like PD, AD, MS, stroke/ ischemia, epilepsy, traumatic brain injury (TBI) and spinal cord injury (SCI), there is a dysfunction of the BBB and BSCB [24]. This dysfunction is characterised by phenotypically altered endothelial cells and decreased tight junction proteins facilitating leukocyte migration [24]. Chronic inflammation or acute injuries can disrupt the integrity of the BBB, leading to the infiltration of systemic immune cells and mediators, further exacerbating a proinflammatory environment in the CNS [24]. Incomplete or impaired responses observed in ageing can be linked to an increased risk of developing neurodegenerative diseases [25]. Innate immune responses recruit cells of the adaptive immune system by secreting various cytokines and chemokines that prompt local cells to express adhesion molecules on the BBB and costimulatory molecules on microglia [23-26].

## 4 NEURODEGENERATION AND NEUROINFLAMMATION

Neurodegeneration occurs when cells of central nervous system discontinue their physiological roles and eventually die, leading to a loss of neural function. It is mediated by the production of neurotoxic intermediaries, which activate inflammatory responses (neuroinflammation) [27]. Neuroinflammation comprises the inflammatory response against abnormal proteins and their aggregates, pathogens and cell danger messengers, and the local activation of glial cells, astrocytes and oligodendrocytes leading to neuronal damage [27-29].

Fig. (**1**) is a schematic representation of the relationship between neurodegeneration and neuroinflammation. Alterations in cell metabolism and/or protein expression can lead to immune response activation. The migration of leukocytes to the CNS is a secondary event after local cell activation.

**Fig. (2):**
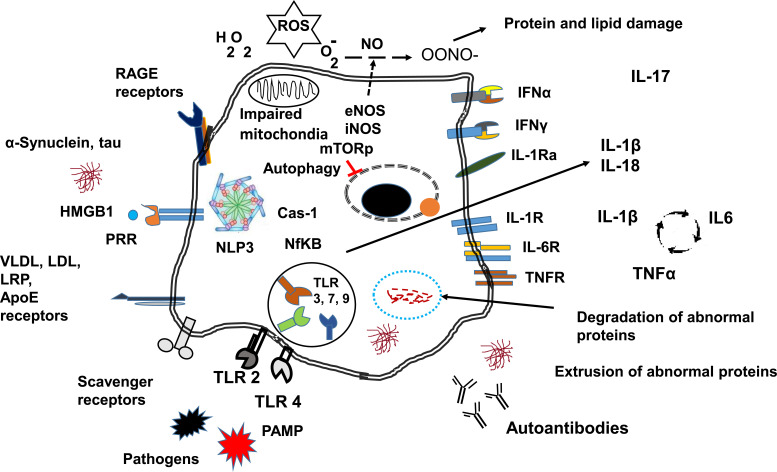
The cellular receptors and pathways involved in neurodegeneration and neuroinflammation. The figure represents the events involved in cell activation in CNS. The processes of cell activation englobe microglia, oligodendrocytes, astrocytes and neurons.

Pathogens and byproducts of cell death, as well as danger signals from damaged or stressed tissues (known as DAMPs), trigger the activation of local central nervous system cells through specialized pattern-recognition receptors (PRRs). Among the proteins involved in danger signals are heat shock proteins, chromatin, high mobility group box chromosomal protein 1 (HMGB-1), and aggregated modified or misfolded proteins such as amyloid-beta (Aβ), α-synuclein (α-syn), and tau. The main PRRs are the Toll-like receptors (TLRs) [29-31]. However, other receptors are also involved in cell activation, such as advanced glycation endproducts receptors (RAGE) [32], VLDL/Apo E, and scavenger receptors that bind apolipoproteins lipoproteins and undegraded products of metabolic pathways (Fig. **2**). Cell activation through these receptors leads to inflammasome activation (NLP3) along with Cas-1 generating IL1β and IL-18 through NFκB activation. NFκB activation induces the transcription of proinflammatory cytokines [31]. The physiological inhibition of IL-1β is IL-1 receptor antagonist (IL-1ra) [33]. This receptor binds IL-1β but does not generate a biological effect [33]. In the absence of IL-1ra, the autocrine effects of IL-1β induce the transcription of IL-6 and TNFα, amplifying the inflammatory response. IFN α is produced upon pathogenic infection and alerts the cells of pathogen invasion [34]. IFNγ receptors are related to microglia activation in pathological conditions. Other cytokines like IL-17 can be produced by activated T lymphocytes recruited by the inflamed tissue, and this cytokine recruits neutrophils to the inflammatory site.

Inflammasome activation has been suggested to induce the onset of AD and PD [34]. Senescence and inflammatory markers may help predict clinical progression in PD patients [35] since the innate immune sensors NLRP3 and Cas-1 are often activated [36]. Dysregulated NLRP3 function observed in aged mice confirms the involvement of NLRP3 in cognitive dysfunction and physical performance; therefore, NLRP3 is an excellent therapeutic target for multiple age-related neurological disorders [37]. The IL-1β signal cascade is an essential pathogenic factor in NDs. Overexpression of caspase-1 and IL-1 β were reported in the nigrostriatal regions of a PD mouse model and the brain and cerebrospinal fluid of PD patients [38, 39]. Blocking IL-1β ameliorated pathological changes in a mouse model of AD [40]. Even though evidence suggests that Caspase-1 may turn α-syn into a highly aggregation-prone variant [41], it is not directly responsible for the death of dopaminergic neurons. Unfortunately, Caspase-1 inhibitors did not improve the survival of grafted dopaminergic neurons in mouse PD models [42]. Activation of TLR due to dysbiosis in the gut microbiome may further impair immunity and accelerate disease progression in PD patients [43]. TLR gene transcription and protein expression are increased in NDs. TLR2 and TLR4 are upregulated in AD [44, 45]; TLR2, TLR5 and CD14 in PD [28, 46]. It is yet unclear whether this over-expression is a compensatory mechanism against the accumulation of toxic proteins and/or alternative cell activation that contributes to disease progression [28, 44], for example, the SNP Asp299Gly polymorphism of the TLR4 gene (linked to susceptibility to gram-negative bacterial infections) attenuates the inflammatory response. That specific polymorphism may protect against sporadic AD [45].

The accumulation of AGEs in cells and tissues is a standard feature of ageing, which is accelerated in neurodegeneration [32]. AGEs are responsible for forming amyloid plaques and neurofibrillary tangles involved in astrocytosis, microgliosis, and neuronal cell death [32]. Activated astrocytes, oligodendrocytes and microglia decrease myelin production and poorly execute repair mechanisms [47, 48]. NFkB plays an essential role in NDs since it is responsible for the transcription of proinflammatory cytokines [49, 50]. Several authors have reported an upregulation in the transcription of beta-amyloid precursor protein cleaving enzyme 1 (BACE1) by NFkB. BACE1 induces beta-amyloid production and the transcription of miR-125b in AD [48, 49]. Dopaminergic neurons in PD brains expressed higher levels of activated NF-κB than controls [50, 51]. Additionally, dysfunctional NFkB cell signalling is involved in neurodegeneration. NFkB promotes the transcription of proinflammatory cytokines [31] and high levels of RelA in the nigral dopamine neurons and glial cells. On the contrary, the c-Rel subunit can exert neuroprotective actions in PD [31]. Uncontrolled TNF-α secretion plays a role in the pathogenesis of neurodegenerative disorders [52, 53]. TNF-related apoptosis-inducing ligand (TRAIL) can be triggered in neurons by β-amyloid and consequently cause apoptosis of brain cells [53]. TRAIL is expressed in the cerebral cortex, often near Congo-red-positive amyloid plaques in the brains of AD patients [54]. Cytokine accumulation in the brain has been observed in PD, ischemia, and AD, leading to chronic inflammation, gliosis, synaptic loss, and glutamate toxicity [55, 56]. In AD patients, increased pro-inflammatory cytokine levels are correlated with low levels of IL-1ra [56]. It has become evident that acute inflammatory responses increase the risk and progression of neurodegenerative diseases; however, controlling inflammation and delivering suitable therapies on time can result in better patient outcomes. Cognitive decline in transgenic AD mouse models was associated with elevated TNF-α levels in the brain. Furthermore, when the TNFR1 gene in transgenic AD mice was deleted, researchers observed an increase in Aβ generation, plaque burden and cognitive deficits [57]. Interestingly, another AD mouse model showed an inverse correlation between cytokines IL-1 and TNF α production and amyloid-β clearance [58]. Astrocytes and oligodendrocytes are affected by increased production of reactive oxygen (ROS) and nitrogen species (RNS). These radicals amplify the inflammatory response [25-36], causing neurodegeneration as observed in experimental models of senile plaques in AD and ALS [59].

The inflammatory response induces changes in BBB and BSCB, facilitating leukocyte migration. TLRs may also play roles in neuronal plasticity as they regulate the processes involved in neurogenesis and neurite outgrowth [24-26, 48]. Inflammaging is a state of chronic low-grade multi-organ inflammation often seen in older adults [59]. Senescent cells, characterised by damaged or weakened cellular repair mechanisms, are responsible for this form of immune dysregulation. Most importantly, “pathogen-free” inflammation may contribute to PD and AD pathogenesis [60]. Indirect evidence of subclinical inflammation was found in the Framingham study, where higher spontaneous production of IL-1 or TNFα (AD markers in older individuals) by peripheral blood mononuclear cells was observed and defined as subclinical inflammation [61]. Under physiological conditions, α-syn, highly expressed in the brain, is involved in lipid metabolism, microtubule activity, modulation of tau phosphorylation and neurotransmitter release [62]. In pathological circumstances, neurons can release α-syn, generating inflammatory responses. Impaired lysosomes cannot degrade internalised α-syn; thus, cathepsin B, a proteolytic enzyme that promotes inflammation [63], is released into the cytoplasm [63-65]. Pathological α-syn becomes insoluble by forming β-sheet-like oligomers (protofibrils) [66]. This insoluble form interacts with microglial TLR2, which activates NF-κB and NLRP3, leading to the microglia's release of TNF-α and IL-1β, causing neuroinflammation in PD [67]. This inflammatory cascade may be further exacerbated by mitochondrial dysfunction, observed in postmortem tissue of PD patients and models [68]. A proposed mechanism for the progressive nature of PD is that misfolded oligomeric α-syn spreads from cell to cell and induces misfolding of native α-syn in a prion-like fashion [69], resulting in the loss of dopamine pathways in the substantia nigra (SN) and the development of Lewy bodies [69, 70]. Impaired dopamine activity is therefore responsible for slow movements (bradykinesia), muscular rigidity, trembling, postural instability, autonomous nervous system alteration and other nonmotor signs such as memory impairment [71]. Increased secretion of abnormal proteins leads to the formation of autoantibodies.

Autophagy is impaired in NDs [72, 73]. The process is essential for cell survival and eliminating unwanted or pathogenic proteins and organelles. The critical sensor of the process is mTOR [72]. In nourished neurons, phosphorylated mTOR blocks autophagy and apoptosis. However, mTOR can be anomalously activated in pathological stressed neurons by radicals, inflammatory intermediates and dysfunctional mitochondria, rendering cells unresponsive [72-74]. Consequentially, cellular metabolic impairment in the CNS leads to neurodegeneration. Thus, metabolic impairment of cells in the CNS is responsible for several processes involved in neurodegeneration.

Several reports have linked lipid metabolism and transport genes with NDs [75]. The involvement of APOE in AD has been extensively analysed. However, due to impaired mitochondria metabolism, lipid degradation is limited, promoting lipid accumulation in droplets. These lipid droplets lead to a more dysfunctional cell [76]. Fatty acid-overloaded astrocytes accumulate acetyl CoA, inducing STAT3 activation [76]. Activated STAT3 activates microglia and downregulates the production of cellular phospholipids and myelin [76]. Dietary supplementation or regular consumption of healthy fats (ω3 fatty acids and short-chain fatty acids) seems to provide a certain degree of neuroprotection and reduce disease progression by lowering inflammation [77, 78] and, perhaps, by improving the composition of the patient’s microbiota [79-80]. A genetic correlation between AD and gut microbiota has recently been published [81]. In the analysis, *Eubacterium fissicatena* was found to be a protective bacteria, while *Collinsella*, and *Veillonella* species were linked to an increased risk. Further research is needed to establish the role of diet and microbiota in neurodegeneration and immune interactions. In the dopaminergic neurons of PD patients and mouse models, the microsomal enzyme prostaglandin E synthase-1 (mPGES-1) was shown to be overexpressed, and consequently, prostaglandin E2 levels were locally increased [75]. Deleting the mPGES-1 gene stopped PGE2 production in these neurons and inhibited neurodegeneration in PD model 6-OHDA [75]. Fig. (**2**) illustrates different cellular receptors and general responses related to neurodegeneration and neuroinflammation. On the left side, various receptors are essential in cell activation and stimulation of the immune response through cytokine secretion and abnormal proteins leading to autoantibody production. In the centre, the rest of the processes are described earlier.

## 5 IMMUNE RESPONSE IN NEURODEGENERATION

Microglia, a type of macrophage exclusive to the CNS, usually produces antiinflammatory molecules and neurotrophic factors (NF) that influence the behaviour of astrocytes and neurons [71]. When these cells encounter pathogens or damaged tissue, they activate, promoting an inflammatory response that engages the immune system and initiates tissue repair. In most cases, this response is self-limiting. In ageing and other conditions such as AD, microglia become hyper-reactive, secreting large amounts of cytokines, chemokines, and other neurotoxic molecules. As expected, microglia have a different transcriptome profile in neurodegenerative diseases and ageing than normal tissue [35-37, 82]. Postmortem tissue from PD and AD patients exhibited HLA-DR^+^ reactive microglia [38, 39], which surges with neuronal degeneration throughout the nigrostriatal pathway in PD [39, 40].

Astrocytes, microglia, and neurons express components of the complement pathway and its receptors [41], suggesting that local inflammation activates the complement cascade activation, leading to cellular stress and death. An increased amount of complement cascade molecules has been reported in the plasma, CSF, and brain tissues of patients with NDs [42]; for example, in PD patients, complement molecules are increased along with inflammatory cytokines [43]. In AD, Aβ plaques are surrounded and infiltrated by activated astrocytes and microglia, which are believed to be the primary source of antigen-antibody-complement complex [83].

Fibrillar Aβ, β-pleated sheets, and tau neurofibrillary tangles have been shown to directly activate the classic complement pathway *in vitro* without antibodies [83]. C1q was found to be tightly associated with Aβ plaques and caused surrounding neuronal atrophy through microglial engulfment [83]. Additionally, C3 production was linked to increased activated NFκB in the brains of AD patients [83-86]. Inhibition of the C1q pathway (using either antibody treatment or gene knockout) in wild-type mice prevented synapse loss after an injection of oligomeric-Aβ [85-87]. Also, C5a receptor inhibitors seem to decrease Aβ plaque load and reduce hyperphosphorylated tau and neuroinflammation in AD mouse models [85, 86].

Neutrophils are essential members of the innate immune response. They release proinflammatory and cytotoxic factors that induce cell death [88, 89]. They enhance cellular metabolism and cytokine-mediated signalling, organise mitochondria, and activate leukocytes in AD patients [89]. These immune cells enter the AD brain *via* LFA-1 integrin and surround Aβ plaques with neutrophil extracellular traps (NET), promoting BBB damage and neuronal toxicity [89]. In contrast, blocking LFA-1 integrin decreases neutrophil population and traffic in AD mouse models, reducing memory loss and neuropathological features [89].

Mast cells are tissue polymorphonuclear cells involved in innate immunity. They are involved in neuroinflammation by inducing oxidative stress, secreting chemokines and inflammatory cytokines, and activating microglia. These cells have been involved in AD and ALS [90].

NK cells are part of the innate immune lymphocytes (ILC) involved in the immunosurveillance of tumours and elimination of senescent cells [90]. Their role in neurodegenerative disease is still under research. Its dysfunction is implicated in infection, malignancy, inflammatory disorders, and age-related senescent cell accumulation [91, 92]. Two NK cell subpopulations are detected in peripheral blood: a) A cytotoxic NK cell that expresses CD16 and is involved in neuroinflammation; b) A tolerogenic NK cell expressing CD56 and assisting in the resolution of neuroinflammation [92]. NK cells have three main cytotoxic mechanisms: 1) the release of granzyme and perforin, 2) activation of the extrinsic apoptotic pathway by Fas ligand and TRAIL, and 3) antibody-dependent cell-mediated cytotoxicity (ADCC) [93, 94]. Alterations in peripheral NK cell number and receptor expression have been reported in PD patients and mouse models [94, 95], yet additional research is needed to understand the role of these cells.

### 5.1 MHC and CD1 in Neuroinflammation

Different HLA genes associated with autoimmune diseases are linked to PD (HLA-DRA and HLA-DRB1) [18, 19]. HLA-DR antigens are upregulated in the microglia of these patients [96]. Interestingly, a genome-wide association was observed between the CD1a gene polymorphism and the increase in neurofilament light in elderly individuals [97]. Neurofilament light is a cytoplasmic protein highly expressed in large myelinated axons in neurological diseases. The migration of CD1a-positive myeloid cells into plaque-associated microglia suggests a link between this antigen and neurodegenerative diseases. CD1a also presents T cells to self and abnormal lipids linked to AD [98]. Dyslipidemia, inflammation and neurodegenerative diseases are connected; however, statin therapies have not been effective, at least in PD [99, 100].

T lymphocytes (Fig. **4**). In NDs, increased amounts of proinflammatory T cells (Th1 and Th17) have been reported, along with decreased numbers of antiinflammatory T helper cells (Th2) and Treg [101-106]. Interestingly, autoimmune-specific T cells were found to facilitate CNS healing processes in sterile mechanical injuries to the brain or spinal cord (Protective autoimmunity). Memory-specific brain self-antigens CD4+ T cells were found in healthy humans and rodents [105, 106]. However, recent AD [101-106] and PD [105-108] data demonstrate that active cytotoxic T cells damage neurons. The apparent contradiction lies in when the cytotoxic cells are detected. At early stages, eliminating neurons that carry pathological protein accumulation may delay disease progression; however, tissue destruction is uncontrolled in later stages as many more cells and processes have been affected.

T cells that recognise Aβ1-42 as an antigen are detectable in AD [109] and likely contribute to forming plaques [110]. AD patients had increased activated CD8+ T cells in the CSF compared to healthy older adults, correlating with clinical and structural AD markers [107]. In AD, CNS infiltrating T cells produce IFN-γ and IL-17, activating microglia and exacerbating neuroinflammation [111-114]. CCL5 (RANTES) is the most common chemokine involved in AD neurodegeneration since it regulates the expression and secretion of normal T cells [111, 115].

In murine models, upregulation of α-syn induces infiltration of B and T lymphocytes in the substantia nigra pars compacta [116-118] and CD3+/CD4+ T cell migration into the neocortex, hippocampus and striatum [117, 118]. However, dopaminergic neurons were spared if the mice were CD4 deficient [119].

Fig. (**3**) depicts the different subpopulations that arise from naïve CD4 and CD8 cells, the cytokine requirements and the production of cytokines. The role of the different CD8 subpopulations is described since it is less common in the literature than CD4 subpopulations.

Lymphocyte-Activation Gene 3 (LAG3) Receptor (CD223) has been recently implicated in PD pathophysiology [120-124]. In a study performed in China, females were more likely to carry variants of this gene [121-142]. Soluble CD223 was considered a prediction marker [123] since it interacts with TLR4, an essential trigger of neuroinflammation in PD [124, 125]. Even though CD223 is involved in T-cell inhibition and exhaustion, its role in PD remains a matter of research.

**Fig. (3):**
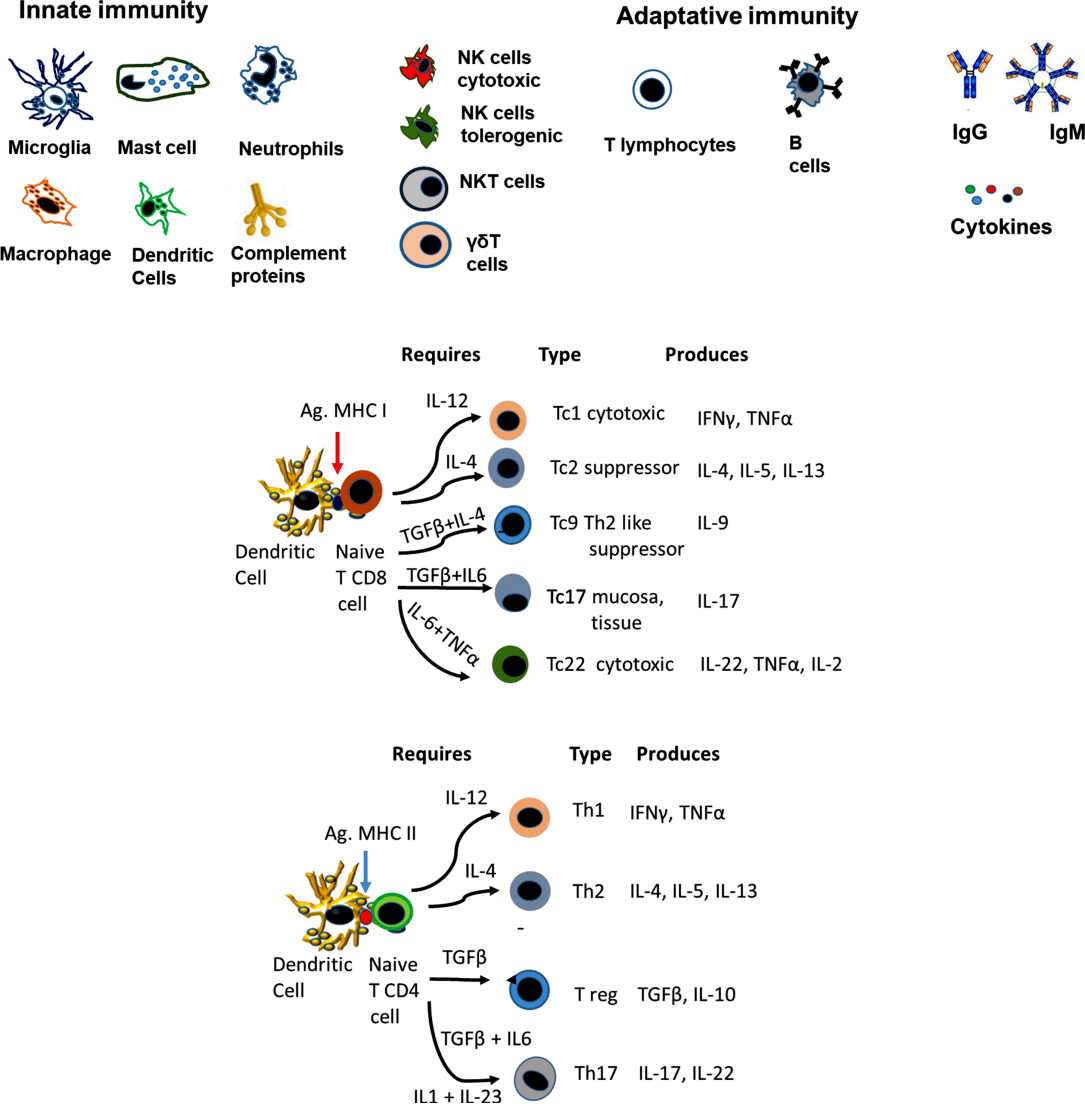
Illustrates the diverse components of the immune response involved in neuroinflammation. (**A**) Three groups are observed. The innate immune response group involves microglia, mastocytes, neutrophils, macrophages, dendritic cells and proteins of the complement cascade. The second group, with innate and adaptative immune response characteristics, are NK, NKT and Tγδ cells. The adaptative group included T lymphocytes, B lymphocytes, antibodies and cytokines. (**B**) illustrates the major T-cell subpopulations that arise depending on the central CD4 and CD8 subpopulation, the antigen presented and the cytokines involved in the differentiation. Cytokines are crucial for the differentiation of T-cell subpopulations, which are involved in physiological and pathological responses.

**Fig. (4):**
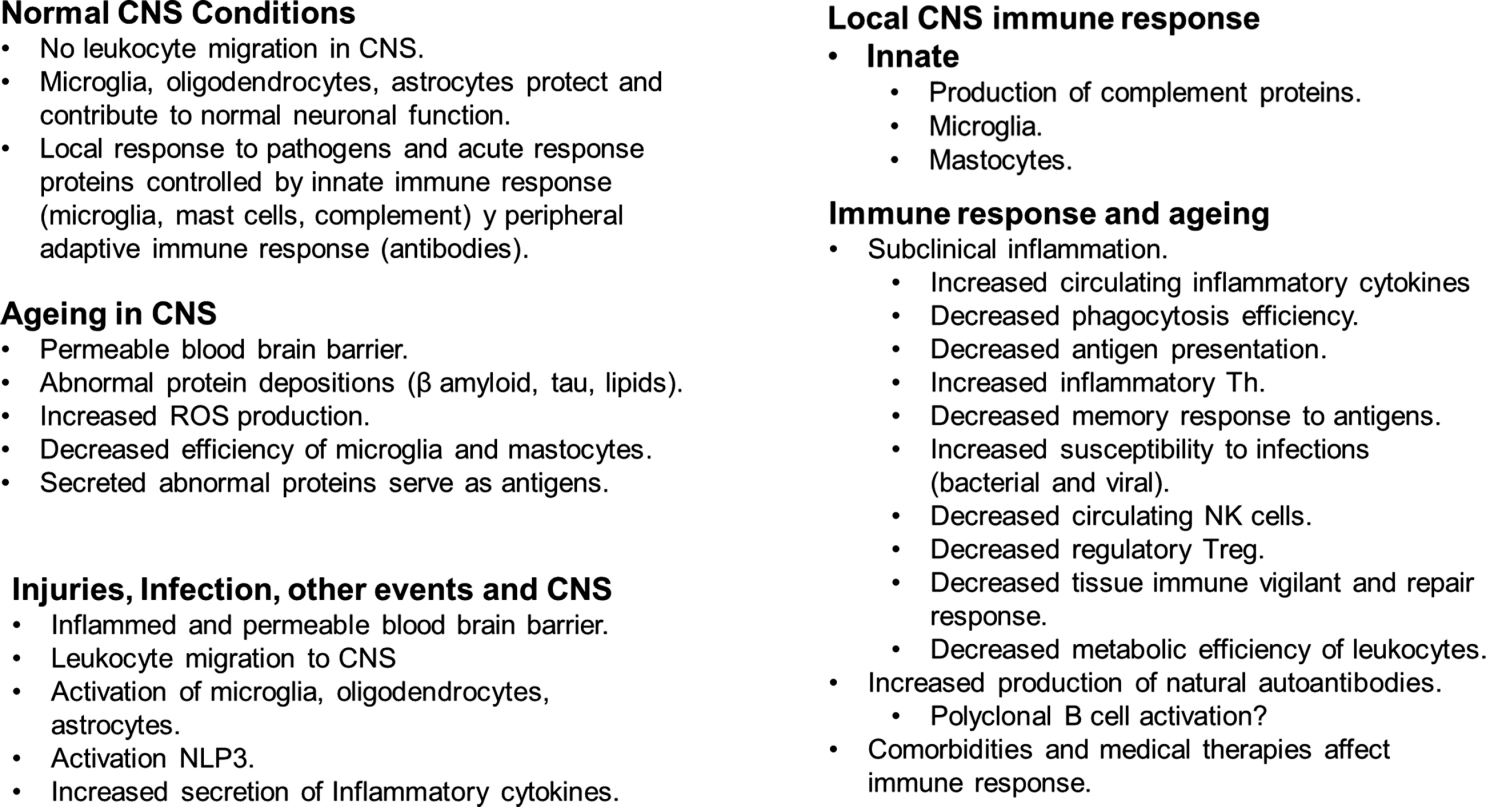
The schematic figure illustrates the main characteristics of immune response in injuries, infection and ageing in the CNS.

Treg (Fig. **3**). These cells provide neuroprotective effects against many neurological diseases in healthy individuals [126]. Tregs can reduce C3 Astrocytes activation and, consequently, inflammation [126, 127] in PD [128] and AD models [129-131]. Specific Aβ1-40 Tregs can prevent the development of Aβ plaques; however, AD patients lack Tregs [132]. Low levels of Foxp3+ regulatory T cells, often reported in females with the Apoeξ4 genotype [131], may affect the choroid plexus in AD [131]. In ALS, these cells are dysfunctional and correlate with disease progression rate and severity [127].

Transferring Tregs to reduce neuroinflammation and promote cell survival has been proposed as a therapeutic strategy for AD [133]. Cognitive abilities improved in mouse models after treatment with Treg-blocked Th1 responses and reversed Aβ-induced inflammation [133]. In turn, higher levels of IL-2, IL-6, TNFα, MCP-1 and T cells were found in PD models [125, 128].

Moore and coworkers [134] showed that vitamin-D induction of T-reg cells in animal models decreased neurodegeneration. The therapeutic use of vitamin D in patients with neurodegenerative disorders has been proposed since many patients usually lack ergocalciferol [135].

Th17 cells (Fig. **3**). These cells were reported to be increased in animal models of neurodegenerative diseases [136]; the IL-23/IL-17A axis has been related to age-associated inflammation. In early PD, circulating Th17 cells augment, some of which respond to α-syn stimulation [137-139]. Genetic variations and microbial infections are primarily responsible for upregulating IL-17A and increasing AD susceptibility. IL-17A also promotes β amyloid production, neutrophil infiltration to the brain, neuroinflammation, increased FASL, and microglial activation [139].

Other T cytotoxic (Tc) subpopulations like Tc9 and Tc22 (Fig. **3**) have been indirectly involved in mouse models of PD and AD; however, more studies are needed to define the role of these diseases and their importance in human pathology.

B cells may be directly involved in ND as they contribute to pathogenesis [140-142]. Although B cells have not been detected in the brains of patients with PD [140, 141], IgG-coating Lewy body [142] deposits are found on dopaminergic neurons, suggesting B cell activation is involved in this pathology. The amount of IgG immunopositive neurons is inversely proportional to the cell loss in the substantia nigra [142]. Most neurons were IgG1-positive, but IgG2 and IgG3-positive neurons were also present, IgG2 being mainly prominent in the damaged substantia nigra [142].

A general summary of the immune response observed in injuries and ageing is presented in Fig. (**4**). The figure aims to give a background of the changes in both conditions and how these changes can be related to NDs. Several characteristics observed in ageing are comparable to those described in NDs.

## 6 AUTOIMMUNITY MARKERS AND NEURODEGENERATIVE DISEASES

The presence of autoantibodies in NDs has been documented. Serum and CSF levels of antibodies against Aβ42 (the most aggregation-prone and neurotoxic species of Aβ) seem to differ between AD patients and healthy controls, but the research is inconsistent [143-145]. Autoantibodies appear to be essential for AD diagnosis [146].

In an early study, autoantibodies against dopaminergic neurons were reported in the CSF of 78% of PD patients compared with 3% of controls [147]. Chen *et al.* [148] demonstrated that plasma antibodies isolated from PD patients induced the loss of dopaminergic neurons in rats. Moreover, the CSF of PD patients showed a cytotoxic effect on dopaminergic neurons, which enhanced SN degeneration in a time- and dose-dependent manner [149]. Multi-epitopic autoantibodies against α-syn were detected in the serum of 65% of all patients with PD [150]; their presence strongly correlated with an inherited mode of the disease but not other disease-related factors. In another study, total autoantibody levels were significantly higher in the PD group than in AD patients and healthy controls [151]. Interestingly, one research group detected reduced α-syn natural autoantibody levels in patients with PD compared to individuals with AD and HC [152]; other groups have reported differences [153, 154]. Autoantibodies against melanin [155], GM1 ganglioside [156] and anti-beta2-glycoprotein I have also been described [157]. There are several possible mechanisms by which autoantibodies may induce dopaminergic cell death [157]: 1) receptor-induced extrinsic apoptosis, 2) antibody-complement complex cell death, 3) activation of surrounding microglia and 4) competitive binding inhibition [157]. In 77 PD patients, Benkler *et al.* [158] found three prevalent autoantibodies: a) antineuronal cells 10.3% *vs.* 1.3% of controls; b) anti-brain lysate 9.1% *vs.* 1.3%; c) anti-dsDNA 10.3% *vs.* 2.6%. Anti-dsDNA was related to dyskinesia, whereas anti-dsDNA and anti-brain lysate were related to depression [158]. Additionally, IgM autoantibodies and anti-myelin-associated glycoprotein (anti-MAG) were significantly elevated in the CSF of PD patients [158, 159]. It is imperative to mention that some autoantibodies have been observed in patients with para-neoplastic syndromes [160], although more research is needed to understand their presence better.

There is a molecular similarity between a protein of herpes simplex virus 1 (HSV1) and human α-syn [161]; autoreactive antibodies produced against HSV1 infection cross-react with a human α-syn homologous peptide. In a serologic study, 58% of PD patients were positive for this protein compared to 18% of controls [161]. This peptide is expressed in the membrane of dopaminergic neurons, leading to immune cell attraction and activation, which later destroys them [161].

Antigenic epitopes can activate CD8^+^ T cells involved in autoimmune responses and may play an important role in neurodegenerative diseases [162]. CD4+ and CD8+ T cells of PD patients recognise α-synuclein peptides [112, 113, 162], and genome-wide association studies have associated PD with MHC genes (HLA-DRA and HLA-DQB1) [18]. Perhaps the thymus lacks α-syn epitopes, and thus, negative selection of T lymphocytes does not occur [163].

Neuromelanin (NM) is another potential target of autoimmune attacks on dopaminergic neurons as DC maturation is triggered upon their recognition [164]. The autoimmune response against NM would be directed against NM-rich cells in the brain, leading to dopaminergic cell death [165]. Unsurprisingly, PD patients were demonstrated to have higher levels of anti-neuromelanin antibodies in serum [164]. Deposits of complement C1q on the surface of extracellular neuromelanin were found in the brains of postmortem PD patients [166, 165].

Frontotemporal lobar degeneration (FTLD) is a neurodegenerative disorder characterised by intracellular accumulation of ALS-related proteins fused in sarcoma (FUS) and TAR DNA-binding protein 43 (TDP43), as well as tau. Behavioural alterations, language impairment, and deficits of executive functions are often observed in FTLD. Reports conclude that 23.4% of FTLD patients had serum autoantibodies against the GluA3 receptor, α-amino-3-hydroxy-5-methyl-4-isoxazole propionic acid receptor (AMPAR) [167, 168]. The incubation of primary cultures of rat hippocampal neurons with anti-GluA3 antibody-containing CSF led to decreased GluA3 subunit synaptic localisation of the AMPA receptor and dendritic spine loss. Antibody titers correlate well with age at disease onset, with earlier symptom onset observed in those patients with higher antibody levels [167-169]. One study observed autoantibodies in 18.9% of patients with degenerative dementia (FTD = 114, AD = 53, and -DLB = 7) [169]. The autoantibodies most frequently detected were 1) the anti-extractable nuclear antibody profile, 2) the rheumatoid factor antibody, 3) the perinuclear antibody and 4) the cytoplasmic anti-neutrophil cytoplasmic antibodies [169]. It is essential to mention that these antibodies are also usually involved in several autoimmune disorders.

High levels of Aβ-IgG immune complexes were found in AD patients' blood serum and CSF and were associated with poor performance on cognitive tests [170]. Moreover, antibodies against AD-related proteins are also increased 1) tau [171], 2) heavy neurofilaments [171], 3) the nicotinic acetylcholine receptor α7 (α7 nAChR)-specifier [172], 4) dopamine [173], 5) serotonin [173], 6) glutamate [174], 7) glutamate receptor [175], 8) S100b (an acidic calcium-binding protein produced by astrocytes) [176, 178], 9) glial fibrillary acidic protein (GFAP) [177], 10) microglia [178], 11) astrocyte autoantibodies [179], 12) oxidised low-density lipoproteins (ox-LDL) [180], 13) rabaptin-5 (a protein involved in cellular vesicle trafficking) [181], 14) the receptor for advanced glycosylation end products (RAGE) [182], 15) angiotensin-2 type-1 receptor [183], 16) aldolase [178], 17) ATP synthase [184], and 18) ceramides [185] (autoantibodies in AD reviewed in [186]).

AD considers Natural autoantibodies against Aβ protective since they assist protein clearance [186]. However, active and passive immunisations with Aβ for therapeutic purposes may lead to immune-complex deposition and perivascular inflammation [186].

Autoantibodies to ATP synthase could be pathogenic in AD since they may inhibit ATP synthesis, alter mitochondrial homeostasis and induce apoptosis [184, 187]. In mice, the intracerebroventricular administration of ATP synthase autoantibodies, purified from AD patients, caused neuronal damage in the hippocampus [188]. Also, autoantibodies to ceramide increased amyloid plaque burden in a transgenic mouse model of AD [189]. Thus, using monoclonal antibodies to decrease the amount of abnormal protein deposition seems to produce pathologic precipitates in the tissue leading to more damage.

## 7 NEURODEGENERATIVE DISEASES IN PATIENTS SUFFERING AUTOIMMUNE DISEASES

The risk of neurodegenerative disease in patients with autoimmune diseases is still an area of intensive research. In Sweden, an analysis involving 310,522 patients and 33 autoimmune disorders showed an increased risk of PD in patients with Graves's disease, Hashimoto's disease, pernicious anaemia, and rheumatic polymyalgia [190]. The same group reported a higher incidence of dementia in patients with type 1 diabetes mellitus, giant-cell arteritis, pernicious anaemia, Sjögren's syndrome, sarcoidosis, celiac disease, chronic rheumatic heart disease, Crohn's disease, chronic glomerulonephritis, pemphigus, psoriasis, rheumatoid arthritis, and ulcerative colitis [191]. In a Korean population-based study, Cho *et al.* [192] showed that Graves' disease patients had a 33% higher risk of developing PD than controls, regardless of age, sex or comorbidities [192]. However, one study did not find a significant difference in the prevalence of thyroid autoimmunity and dysfunction between PD patients and neurological controls (10.8% in PD patients *vs.* 10% in neurological controls) [193]. These results were later confirmed in a meta-analysis [194]. In a Mendelian randomisation study, multiple sclerosis and Sjögren syndrome were more strongly associated with AD than psoriasis, rheumatoid arthritis (RA) and type 1 diabetes [195]. Epidemiological, genetic and clinical research is required on this topic.

There are still controversies concerning the possible risk of neurodegenerative diseases in patients with RA. Some groups have shown an increased risk of dementia [196-198], while others have not [199, 200]. On the other hand, Policicchio *et al.* [201] demonstrated a lower incidence of AD in RA patients [201]. The discrepancies in interpretation may rely on the monitorisation of the inflammatory condition. Cooper and coworkers [202] showed a correlation between C reactive protein levels, RA and increased risk of PD [202], suggesting that chronic inflammation brought on by the disease may lead to the development of NDs. There is a higher prevalence of PD in patients with bullous pemphigoid, an autoimmune blistering dermatosis of elders, compared to patients with psoriasis [203]. Further, patients with ankylosing spondylitis are at higher risk of AD and PD [204].

Inflammatory bowel disease, IBD (Crohn's disease and ulcerative colitis), was identified as an independent risk factor for PD and AD development [205-207]. In a Danish study, IBD was associated with slightly increased dementia risk, particularly FTLD [205]. Similar results were found in a Taiwanese study [206]. One meta-analysis revealed a higher risk of AD and PD among Crohn’s disease and ulcerative colitis patients [207]. Another exciting report by Aggarwal *et al.* [208] showed that IDB patients manifested AD at younger ages and, in addition to IBD, other inflammatory poly arthropathies and systematic connective tissue disorders (psoriasis, rheumatoid arthritis and multiple sclerosis) are also linked to AD [209, 210]. In a Mendelian randomisation study, Cui and coworkers [210] found that individuals with IBD had a significantly higher risk of developing PD. However, other Mendelian randomisation studies showed no evidence of an association between IBD and PD [211, 212]. There are still many questions to answer on this topic, as research is quite contradictory.

In Taiwan, Lui FC and coworkers [213] reported an inverse association between systemic lupus erythematosus (SLE), a chronic, systemic autoimmune disease, and the risk of PD, with the crude hazard ratio (HR) being 0.60 (95% confidence interval 0.45-0.79) in comparison with non-SLE patients in a population-based study. Nonetheless, systemic lupus erythematosus and Sjögren syndrome were highly associated with dementia risk in a study by Wang and coworkers [214]. Yet, there wasn’t a significant causal association between SLE and AD in another Mendelian randomisation study [215].

The association between autoimmunity and neurodegenerative diseases is still an evolving topic. In the next section, the effect of therapies that modulate the immune response in autoimmune diseases has generated new perspectives which are essential to analyse.

## 8 IMMUNOLOGIC TREATMENT AND RISK OF NEURODEGENERATIVE DISEASE

In animal models of PD, a reduction in dopaminergic neuron degeneration has been observed in animals treated with nonsteroidal antiinflammatory drugs (NSAIDs) [216]. In two prospective studies (men Health Professionals Follow-up Study,1986-2000, and Women Nurses' Health Study, 1980-1998), a lower risk (0.55) of PD onset was found in the participants who reported regular use of nonaspirin NSAIDs as compared to the non-regular users [217]. In addition, a lower, but not highly significant, risk of PD was also observed among men and women who took two or more aspirin tablets daily compared with nonusers [217, 218]. In another cohort of men and women from the US (The Cancer Prevention Study II Nutrition Cohort), PD risk was lower among ibuprofen users than nonusers [219]. Compared with nonusers, the relative risks were 0.73% for people who consumed fewer than two tablets/per week and 0.62% for those who had one or more tablets/per day [216-218]. Gao and coworkers [219] reported an association between ibuprofen and lower PD risk, not shared by other NSAIDs or acetaminophen. In another study (Neuro Genetics Research Consortium), smoking, coffee, and over-the-counter NSAID use as individual factors exhibited a 20% to 30% risk reduction for PD [220]. Multi-analysis associated the leucine-rich repeat kinase--2 gene penetrance with NSAID use and PD [221]. However, recent meta-analyses had contrasting results for the same disease [222, 223]. Table **1** illustrates the effect of different compounds with anti-inflammatory effects.

Patients with RA treated with TNF-blocking agents (etanercept, adalimumab, infliximab) rarely develop AD [224, 225]. In addition, TNF inhibitors showed a long-term effect in reducing the risk of AD during 20 years of follow-up in RA patients [226]. On the other hand, Etanercept and Adalimumab were associated with lower AD risk in patients with psoriasis [225, 226]. Methotrexate may also have neuroprotective effects [227]. Similarly, the benefit of anti-TNF therapy was observed in patients with ankylosing spondylitis [228]. On the contrary, no effect was seen in patients using conventional disease-modifying antirheumatic drugs (cDMARD) [228]. One study reported a 78% reduction in the incidence rate of PD among patients with inflammatory bowel disease exposed to anti-TNF therapy compared with those not [229].

A lower risk for AD was also associated with using methotrexate combined with anti-TNF [230]. Although, there was no significant difference comparing the risk of AD between RA patients receiving Methotrexate or TNF blockers, only a combination of both [230]. Treatment with abatacept (T-cell activation inhibitor) plus tofacitinib (JAK inhibitor) and tocilizumab (IL-6 inhibitor), or TNF inhibitors, did not decrease the risk of AD in arthritic patients [231]. Studies revealed no statistical association between Alzheimer’s disease and hydroxychloroquine use [232, 233]. A recent publication showed the contrary, with a lower AD incidence risk than methotrexate [234]. Other immune-modulating drugs like sulfasalazine have been linked to neuropathic pain and migraine but not dementia [235]. Further evidence is required.

Plasma levels of microRNA-153, microRNA-223 and microRNA-30e, involved in NLRP3 antagonism, are decreased in PD patients [236]. In particular, microRNA-30e, a negative NLRP3 regulator, reduces the loss of dopaminergic neurons and improves motor and behavioural symptoms [236, 237]. Thus, miRNA-30e may be the therapeutic link between autoimmunity and neurodegeneration.

A decreased incidence of AD has also been observed with other treatments used in autoimmune diseases. Patients treated with calcineurin inhibitors who underwent a solid organ transplant have a lower incidence of AD than the general population [238]. Diagnoses of AD were reduced among individuals ≥ 65 years with prior influenza vaccination compared to those without the vaccine [239]. In a group of patients with bladder tumours, age ≥ 75 years, those treated with intravesical Bacillus Calmette-Guerin (BCG) had a significantly decreased risk of developing AD and PD as compared to patients who only underwent transurethral resection [240]. Other drugs like metformin are still under discussion [241]. Epidemiological analysis of large-scale populations may provide more associations since the above publications could only represent random and unspecific associations when large trials or studies are conducted.

## 9 IMMUNOPHARMACOLOGY AND NEURODEGE-NERATIVE DISEASES

Studies on inflammation, autoimmunity, and neurodegenerative diseases have opened new therapeutic options in neurodegenerative diseases. *In vitro*, releasing toxic factors by activated microglia can be partially blocked by NSAIDs [242].

To facilitate the analysis of different strategies that have been used in PD and AD, we divided the most relevant ones into tables. Table **1** [243-277] represents tested anti-inflammatory compounds, mainly in animal models. The analysis of NSAID, as commented before, was tested in two clinical trials in which no reports have been published suggesting its lack of effect on AD. It should be noted that the population study involved normal individuals in which the risk of NDs was analysed over time compared with the drug's effect on individuals with the incipient disease. The use of anti-tumour drugs is exciting since it may provide new options for patients with known genetic risks for the disease. The rest of the compounds have not reached clinical trials, but chemical modifications may lead to exciting structures with potential use in NDs.

A fascinating approach based on different populations' diets and natural remedies has identified several natural compounds. The primary goal is to decrease oxygen and nitrogen radical formation and discover new anti-inflammatory structures that could pass the blood-brain barrier. Oral intake of some of these compounds has been proven to reduce ND onset and progression. Most of these compounds are flavonoids that significantly affect immune response in several diseases [278].

Different structures used to treat various diseases were repurposed for NDs. Three critical pathways were targeted: NLP3/Cas-1, TNFα inhibition, immunomodulation, p38αMK2 and the aryl hydrocarbon receptor (Table **1**). Promising compounds in preclinical studies underwent clinical trials with mixed results. Neflamapimod seems to be the most promising compound, although more clinical trials and long-term follow-up are required [273, 274]. Laniquimod was previously used to treat MS, is now used to treat HD and may have a significant role in synucleinopathies by decreasing neuroinflammation [275]. Table **2** illustrates the effect of different natural products tested in AD and PD, Curcumin also seems to activate the aryl hydrocarbon receptor, reducing neuroinflammation [309].

As described earlier, complement is produced by different cells in the CNS and autoantibodies against the abnormal proteins have been detected [309]. Therefore, cell death due to antibody complement complex can be blocked by know inhibitors. Even though results in animal models seem promising, there has been only one clinical trial involving neuromyelitis optica, and no other trials have been proposed. This therapy may be used in combination with others as a coadjuvant.

The use of cytokine inhibitors in NDs is an exciting approach (Table **3**); however, the main problem is treating patients with symptoms, especially during the early phases. TNFα inhibitors, also used in autoimmune diseases, have been reported the most. The use of other inhibitors is still under scrutiny (Table **3**). Inhibitors of other cytokines, IL-1 receptor antagonists and IL-12/IL-23 have been analyzed (Table **3**). The possible role of IL-10 is under discussion [326].

Anti-Aβ antibodies in healthy individuals were the basis for clinical trials of intravenous immunoglobulin (IVIg) in patients with AD. However, despite promising initial results, a recent meta-analysis of blood derivatives showed no clear benefit of IVIg after five clinical trials despite promising initial results [327]. No current clinical trials involve IVIg in neurodegeneration.

The use of monoclonal antibodies in AD has gained attention in recent years. After the lack of effect of the first monoclonals, several new schemes for generating new antibodies were used (Table **4**). The more successful ones are Aducanumab and Lecanemab, two antibodies approved by the FDA for AD. Aducanumab has not significantly improved cognitive response in AD patients (Table **4**). Nevertheless, there have severe concerns with Lecanemab about brain shrinkage and patient death. This high-affinity antibody can probably activate damaged cell death, decreasing brain volume [348]. It may be helpful to analyse brain autopsies in detail and the mechanism of this effect before discontinuing their use in the clinic.

Considering that autoantibodies are usually produced against abnormal or phosphorylated tau, it is an excellent strategy to immunize against abnormal tau to prevent the effect of this protein on healthy tissues (Table **5**). The vaccine would require the activation of specific non-polyclonal B cells. It is still early to analyze the first vaccine's impact; nonetheless, several other vaccines are underway, which may be interesting to compare.

The use of monoclonal antibody therapies against α synuclein has not been, up to date, thriving despite the different types of antibodies generated against the variety of pathological proteins (Table **6**). Moreover, vaccine trials are still underway, and it is too early to state whether they are effective (Table **7**).

Several other approaches have been proposed and are underway to perform trials using specific immunization [374-376]. However, care must be taken due to inconveniences reported in previous efforts [377-380].

## 10 OTHER TREATMENTS

Treatments with cytokines that downmodulate inflammatory cytokines and cell activation have also been proposed. One example is the granulocyte-macrophage colony-stimulating factor (GM-CSF). In the PD mouse model, GM-CSF treatment generated a protective Treg response by downregulating microglial activation and decreasing the death of dopaminergic neurons [381]. Sargramostim (GM-CSF) demonstrated a safe and well-tolerated profile. In phase I clinical trials with PD patients, NCT03790670 sargramostim increased Treg frequencies and function without affecting the levels of effector T cells [381]. Compared with pretreatment baselines and placebo-treated controls, sargramostim-treated patients had lower clinical ratings of disease severity, and magnetoencephalography revealed improved signalling in cortical regions relevant to motor function [382]. Five patients with Parkinson's disease who were administered sargramostim for a duration of one year experienced a reduction in Movement Disorder Society-Sponsored Revision of the Unified Parkinson's Disease Rating Scale (MDS-UPDRS) scores [383]. A new clinical trial, NCT05677633, on biomarker validation following sargramostim treatment is underway.

Therapeutic plasma exchange (TPE) plasmapheresis reduces the concentration of pathology-related contents in plasma. TPE has been used in AD [384] and may benefit patients by an entirely different mechanism, potentially opening a new avenue for future research [385]. A phase 2b/3 Alzheimer's Management by Albumin Replacement

(AMBAR) study shows that TPE with albumin exchange may slow cognitive and functional decline in AD patients [385]. A significant improvement in quality of life was measured by a self-reported questionnaire among patients with mild AD from baseline to 14 months among the TPE-treated groups compared with the control group. There are still several areas in this topic that require more research.

Among other strategies proposed is using small molecules to target checkpoint receptors in neuroinflammatory diseases [386]. Also, the inhibition of the pathway IL-17/TRAF6 as this pathway is involved in neurotoxicity [387]. Finally, the use of therapies to expand Treg cells seem to be important not only in MS but also in PD [388]. The development of new treatments is just beginning.

## CONCLUSION

Various schemes involving AD and PD have been used to control acute and/or chronic inflammatory responses to decrease the risk or slow the progression of neurodegenerative diseases. In both diseases, there is still room for improvement. Several therapies for autoimmune diseases have been proven helpful in the onset or progression of NDs. Several anticancer drugs may be beneficial, as in the case of methotrexate in rheumatoid arthritis. The critical issue is assessing the risk and diagnosing the condition in time to start with good therapeutic schemes involving balanced nutrition, supplementation, and physical and cognitive exercises (https://www.alz.org/alzheimers-dementia/treatments/alternative-treatments). Genetic counselling of families of patients with NDs may help identify those with higher genetic risk and provide alternatives to delay disease onset.

Currently, safe therapeutic options involve cytokine inhibitors and other anti-inflammatories in patients with stable disease or typical progression. Monoclonal antibodies against βA must be closely monitored due to their adverse effect.

In patients with rapid progression, there is no primary option available. However, clinical trials should consider this group as more people develop NDs at younger ages.

Reducing inflammation with an array of early-stage treatments is the most promising strategy to mitigate the development of underlying AD and PD pathophysiology [356]. There is, however, room for improvement in pathological screening and the generation of new therapeutic compounds, as well as strategies and schemes that can benefit these highly prevalent diseases.

## Figures and Tables

**Fig. (1):**
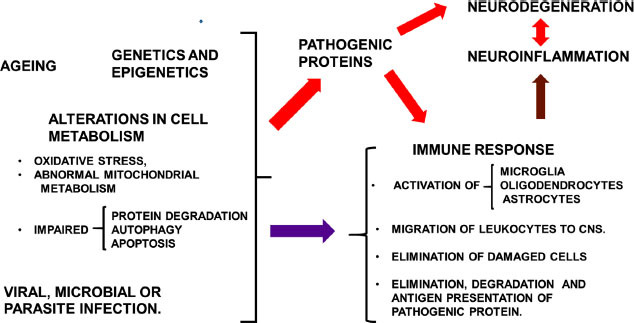
General overview of the interaction between neurodegenerative diseases, immune response, neuroinflammation and neurodegeneration. On the left side, ageing, genetic and epigenetic factors, and viral, microbial or parasite infections induce alterations in cell metabolism with impaired abnormal protein degradation, autophagy and apoptosis, and the secretion of pathogenic proteins (red arrow). Pathogenic proteins can induce neurodegeneration and immune response activation (red arrows). All the previous events can cause activation of the immune response (purple arrow). Local and/or peripheral immune response activation induces neuroinflammation (brown arrow), leading to neurodegeneration. Neural cell death also activates the immune response (red double arrows).

**Table 1:** Effect of different compounds tested in AD and PD.

**Compound**	**Proposed Effect/Mechanism**	**Disease**	**Animal Model**	**Clinical Trial**	**References**
NSAID Ibuprofen Prednisone Fenamic Acid	Anti-inflammatory	AD/PD	Yes	Yes (no effect reported), NCT00239746, NCT00000178	[221, 222, 242-244]
Anti-tumour drugs	Decreases microtubule and Tau phosphorylation	AD	No (*in vitro*)	No	[245-248]
Minocycline	Antibiotic, anti-inflammatory	AD/PD	Yes	Yes, PD (no change) NCT00063193 NCT00076492.	[249, 250]
Sitagliptin Saxagliptin Vildagliptin Exenatide Liraglutide	Antidiabetic	AD	Yes	No	[251-253]
Rosiglitazone Pioglitazone	Anti-inflammatory	AD/PD	Yes	No	[254-257]
TAK-242	Inhibits TLR4	AD/PD	Yes	No	[68, 258, 259]
Baicalin	Inhibits TLR4 and NFkB	AD/PD	Yes	No	[260]
GX50	Inhibits NFκB and MAPK	AD/PD	Yes	No	[261]
AntiTLR2 monoclonal	Inhibits TLR2	PD	Yes	No	[262]
Ibrutinib	Inhibits BTK and NLP3/Cas-1 signalling	AD	Yes	No	[263]
Thalidomide	Inhibits TNFα and acts as an immunomodulator	AD	Yes	No (no effect in preliminary experiments)	[264-265]
Lenalidomide, Pomalidomide	Inhibits TNFα and acts as an immunomodulator	AD/PD	Yes	Yes NCT04032626 (AD)	[266-267]
Cyclosporin	Blocks calcineurin decreases α-syn	PD	Yes	No	[268]
Tacrolimus	Inhibits leukocyte activation and TNFα signalling	AD/PD	Yes	No	[269-270]
GPI-1485 (modified Tacrolimus)	Inhibits leukocyte activation and TNFα signalling	PD	Yes	Yes NCT00076492	[271]
Sinomenine	Inhibits p38α, NFkB and MK2 axis (*in vitro*)	AD/PD	Yes	No	[272]
Neflamapimod	Inhibits p38α and MK2 axis	AD/PD	Yes	Yes NCT03402659 NCT03435861 NCT04001517 (Lewy bodies)	[273, 274]
Laniquimod	Inhibits aryl hydrocarbon receptor	PD	Yes	Not yet	[275]
MW150, MW100	Inhibits p38α and axis MK2	AD	Yes	Yes NCT05194163	[276]
Small synthetic molecules	Inhibits p38α and axis MK2	AD/PD	Yes	Not yet	[277]

**Table 2:** Effect of different natural products tested in AD and PD.

**Compound**	**Proposed Effect/Mechanism**	**Disease**	**Animal Model**	**Clinical Trial**	**References**
Vinpocetine (alkaloid)	TLR modulator. Decreases TLR2 and TLR4 transcription	PD	Yes	Local non-registered 2019	[279]
Farrerol	Inhibits TLR4 and TLR4 pathways	PD	Yes	No	[280]
Kaempferol	Inhibits TLR4 and TLR4 pathways	PD	Yes	No	[281]
Dihydrotestosterone	Inhibits TLR4-induced inflammation	LPS neuro inflamed	Yes	No	[282]
Silymarin (flavonoid)	Downregulates TLR4 expression	PD	Yes	No	[283]
MCC950, Kaempferol, Oridonin, Fingolimod, AZ11645373, Celastrol	Inhibits NLP3	PD	Yes	No	[284]
Arglabin	Inhibits Cas-1 and NLP3 (*in vitro*)	AD	No	No	[285]
Tetrandrine	Inhibits NFkB (*in vitro*)	AD	Yes	No	[286]
Tiliroside, Siliroside	Inhibit NFkB, p38MAPK signalling (*in vitro*)	AD	No	No	[287-288]
Apigenin, Luteolin	Inhibit IFNγ *via* STAT1 (*in vitro*)	AD	Yes	No	[289-290]
Quercetin, Epigallocatechin/3	Inhibit NFkB	AD	Yes	No	[291-293]
Resveratrol	Inhibits PGE_2 _and IL1β	AD	Yes	Yes	[293-294]
Curcumin	Inhibits NFkB and MAPK signalling. Increases anti-inflammatory cytokines through SOCS	AD	Yes	Yes NCT01001637 NCT00099710 No results posted	[295-300]
Parthenolide, Artemisin	Downregulate IL6 and TNFα (*in vitro*)	AD	Yes	No	[301-302]
Thymoquinone, Carnosic Acid, Carnosol, Ginkgolides	Inhibit pre-inflammatory cytokines (*in vitro*)	AD/PD	No	No	[303-305]
Crocin, Crocetin	Inhibit pro-inflammatory cytokines, NO and ROS. NFkB. Induce clearance of βA by autophagy	AD/PD	Yes	No	[306]
Astaxanthin	Inhibits NO, COX/2, and IL6. Induces clearance of βA by autophagy	AD/PD	Yes	No	[307]

**Table 3:** Effect of inhibitors of the complement pathway and cytokines in AD.

**Compound**	**Proposed Effect/Mechanism**	**Disease**	**Animal Model**	**Clinical Trial**	**References**
Eculizumab	Anti-C5 monoclonal antibody blocks cleavage	AD	Yes	Yes, NCT00904826 (Neuromyelitis optica)	[310]
Compstatin family (Cp40 and MNY10)	Inhibit C3	AD	Yes	No	[311]
ANX005	Monoclonal antibody inhibits C1q binding/activity	AD	Yes	No	[312]
Anakinra	IL1 receptor antagonist	AD	Yes	No	[313, 314]
TNFα inhibitors Infliximab Etanercept	Reduce amyloid plaques and Tau `phosphorylation	AD	Yes	Yes NCT01068353 NCT00203359 NCT00203320 NCT04571697 (TNF inh *vs*. methotrexate) Others ongoing	[315-322]
IL-12 and IL-23 inhibitors	Inhibit IL-12 and IL-23	AD	Yes. Gender differences	No	[66, 323-325]

**Table 4:** Monoclonal antibody therapy in AD.

**Monoclonal**	**Target**	**Animal Model**	**Clinical Trial**	**Effect(s)**	**References**
Bapineuzumab	β-amyloid	Yes	Yes	None	[328-329]
AAB-003 (modified Bapineuzumab)	β-amyloid	Yes	Yes	None	[330]
Solanezumab, Ganteberumab	β-amyloid	Yes	Yes	None	[331-334]
Crenezumab	β-amyloid	Yes	Yes	None	[335]
PBD-C06	pGlu-Aβ	Yes	No	Unknown (in humans)	[336]
Donanemab	β-amyloid	Yes	Yes (early AD) NCT03367403 NCT02624778	Cognitive improvement	[337-339]
Aducanumab	β-amyloid aggregates	Yes	Yes (FDA approved) NCT02484547 NCT02477800 Other trials are active	Cognitive improvement	[340-344]
Lecanemab	β-amyloid aggregates	Yes	Yes (FDA approved) NCT01230853 Other trials are active	Improvement of clinical symptoms. Secondary effects?	[345-347]

**Table 5:** Immunization of Tau or antigenic peptides.

**Vaccine**	**Target**	**Animal Model**	**Clinical Trial**	**Effect(s)**	**References**
AAD vac1	Tau phosphorylation	Yes	Yes NCT02031198 NCT02579252 NCT01850238	Lower hippocampal atrophy (less cognitive decline)	[349-354]
Others	Tau phosphorylation/aggregates	Yes	No	Unknown	[355]

**Table 6:** Monoclonal antibodies against α-synuclein explored in animal models of PD and one clinical trial.

**Vaccines**	**Target**	**Animal Model**	**Clinical trial**	**Effect(s)**	**References**
Prasinezumab	α-syn	Yes	Yes	No effect NCT03100149	[356-357]
Cinpanemab	Aggregated α-syn	Yes	Yes NCT03318523	None	[358-359]
MEDI1341	α-syn in the brain	Yes	No	Inhibits the spread of α-syn in mice	[360]
Lu AF82422	α-syn	Yes	No	Unknown	[361]
Rec47	Oligomeric α-syn	Yes	No	Unknown	[362-365]
ABBV-0805	Aggregated α-syn	Yes	No	Decreases α-syn aggregates in mice brains	[365-66]

**Table 7:** The α-synuclein vaccines that have been tested.

**Vaccines**	**Target**	**Animal Model**	**Clinical Trial**	**Effect(s)**	**References**
AFFITOPE^®^ peptides	α-syn epitopes	Yes	Yes (subcutaneous) NCT01885494 NCT02267434	Cognitive improvement	[367]
UB-312	Oligomeric and fibril protein	Yes	Yes (ongoing) NCT05634876	Unknown (still underway)	[368-369]
C-type lectin receptor vaccine	α-syn	Yes	No	Unknown (still underway)	[370]
DNA vaccine	α-syn	Yes	No	Not effective	[371, 372]
MultiTEP	α-syn	Yes	No	-	[373]

## LIST OF ABBREVIATIONS

AD: Alzheimer's Disease
ADCC: Antibody-dependent Cell-mediated Cytotoxicity
ALS: Amyotrophic Lateral Sclerosis
anti-MAG: Anti-myelin-associated Glycoprotein
APP: Amyloid Precursor Protein
Aβ: Amyloid-beta
BBB: Blood-brain Barrier
BCG: Bacillus Calmette-guerin
BSCB: Blood-spinal Cord Barrier
C9orf72: Chromosome 9 Open Reading Frame 72
Cas-1: Caspase 1
CNS: Central Nervous System
DAMPs: Damaged or Stressed Tissues
FTLD: Friedreich Ataxia, Frontotemporal Lobular Degeneration
FUS: Fused in Sarcoma
GM-CSF: Granulocyte-macrophage Colony-stimu-lating Factor
HD: Huntington's Disease
HMGB-1: High Mobility Group Box Chromosomal Protein 1
HSV1: Herpes Simplex Virus 1
IBD: Inflammatory Bowel Disease
MDS-UPDRS: Movement Disorder Society-sponsored Revision of the Unified Parkinson's Disease Rating Scale
MS: Multiple Sclerosis
ND: Neurodegenerative Diseases
NFTs: Neurofibrillary Tangles
NSAID: Non Steroid Inflammatory Drug
PD: Parkinson's Disease
PRRs: Pattern-recognition Receptors
PSEN: Presenilin
RAGE: Advanced Glycation Endproducts Receptors
SCI: Spinal Cord Injury
SMA: Spinal Muscular Atrophy
SNP: Single Nucleotide Polymorphisms
SOD1: Superoxide Dismutase 1
TBI: Traumatic Brain Injury
TDP-53: TAR DNA‐binding Protein
TLRs: Toll-like Receptors
TPE: Therapeutic Plasma Exchange
TRAIL: TNF-related Apoptosis-inducing Ligand
α-syn: α-Synuclein

## References

[1] Price D.L., Sisodia S.S., Borchelt D.R. (1998). Genetic neurodegenerative diseases: the human illness and transgenic models.. Science.

[2] MacDonald M. (1993). A novel gene containing a trinucleotide repeat that is expanded and unstable on Huntington’s disease chromosomes.. Cell.

[3] Akçimen F., Lopez E.R., Landers J.E., Nath A., Chiò A., Chia R., Traynor B.J. (2023). Amyotrophic lateral sclerosis: Translating genetic discoveries into therapies.. Nat. Rev. Genet..

[4] Papiri G., D’Andreamatteo G., Cacchiò G., Alia S., Silvestrini M., Paci C., Luzzi S., Vignini A. (2023). Multiple sclerosis: Inflammatory and neuroglial aspects.. Curr. Issues Mol. Biol..

[5] Klotz L., Antel J., Kuhlmann T. (2023). Inflammation in multiple sclerosis: Consequences for remyelination and disease progression.. Nat. Rev. Neurol..

[6] Balcerac A., Louapre C. (2022). Genetics and familial distribution of multiple sclerosis: A review.. Rev. Neurol..

[7] Breijyeh Z., Karaman R. (2020). Comprehensive review on Alzheimer’s disease: Causes and treatment.. Molecules.

[8] Rizek P., Kumar N., Jog M.S. (2016). An update on the diagnosis and treatment of Parkinson disease.. CMAJ.

[9] Aborode A.T., Pustake M., Awuah W.A., Alwerdani M., Shah P., Yarlagadda R., Ahmad S., Silva C.I.F., Chandra A., Nansubuga E.P., Abdul-Rahman T., Mehta A., Ali O., Amaka S.O., Zuñiga Y.M.H., Shkodina A.D., Inya O.C., Shen B., Alexiou A. (2022). Targeting oxidative stress mechanisms to treat Alzheimer’s and Parkinson’s disease: A critical review.. Oxid. Med. Cell. Longev..

[10] Gorlé N., Van Cauwenberghe C., Libert C., Vandenbroucke R.E. (2016). The effect of aging on brain barriers and the consequences for Alzheimer’s disease development.. Mamm. Genome.

[11] Dai M.H., Zheng H., Zeng L.D., Zhang Y. (2018). The genes associated with early-onset Alzheimer’s disease.. Oncotarget.

[12] Sumirtanurdin R., Thalib A.Y., Cantona K., Abdulah R. (2019). Effect of genetic polymorphisms on Alzheimer’s disease treatment outcomes: An update.. Clin. Interv. Aging.

[13] Sarnowski C., Ghanbari M., Bis J.C., Logue M., Fornage M., Mishra A., Ahmad S., Beiser A.S., Boerwinkle E., Bouteloup V., Chouraki V., Cupples L.A., Damotte V., DeCarli C.S., DeStefano A.L., Djoussé L., Fohner A.E., Franz C.E., Kautz T.F., Lambert J.C., Lyons M.J., Mosley T.H., Mukamal K.J., Pase M.P., Portilla Fernandez E.C., Rissman R.A., Satizabal C.L., Vasan R.S., Yaqub A., Debette S., Dufouil C., Launer L.J., Kremen W.S., Longstreth W.T., Ikram M.A., Seshadri S. (2022). Meta-analysis of genome-wide association studies identifies ancestry-specific associations underlying circulating total tau levels.. Commun. Biol..

[14] Su F., Bai F., Zhang Z. (2016). Inflammatory cytokines and Alzheimer’s disease: A review from the perspective of genetic polymorphisms.. Neurosci. Bull..

[15] Ulhaq Z.S., Garcia C.P. (2020). Inflammation-related gene polymorphisms associated with Parkinson’s disease: An updated meta-analysis.. Egypt. J. Med. Hum. Genet..

[16] Li X., Zhang D.F., Bi R., Tan L.W., Chen X., Xu M., Yao Y.G. (2023). Convergent transcriptomic and genomic evidence supporting a dysregulation of CXCL16 and CCL5 in Alzheimer’s disease.. Alzheimers Res. Ther..

[17] Pedersen C.C., Lange J., Førland M.G.G., Macleod A.D., Alves G., Maple-Grødem J. (2021). A systematic review of associations between common SNCA variants and clinical heterogeneity in Parkinson’s disease.. NPJ Parkinsons Dis..

[18] Hollenbach J.A., Norman P.J., Creary L.E., Damotte V., Montero-Martin G., Caillier S., Anderson K.M., Misra M.K., Nemat-Gorgani N., Osoegawa K., Santaniello A., Renschen A., Marin W.M., Dandekar R., Parham P., Tanner C.M., Hauser S.L., Fernandez-Viña M., Oksenberg J.R. (2019). A specific amino acid motif of HLA-DRB1 mediates risk and interacts with smoking history in Parkinson’s disease.. Proc. Natl. Acad. Sci..

[19] Yu E., Ambati A., Andersen M.S., Krohn L., Estiar M.A., Saini P., Senkevich K., Sosero Y.L., Sreelatha A.A.K., Ruskey J.A., Asayesh F., Spiegelman D., Toft M., Viken M.K., Sharma M., Blauwendraat C., Pihlstrøm L., Mignot E., Gan-Or Z. (2021). Fine mapping of the HLA locus in Parkinson’s disease in Europeans.. NPJ Parkinsons Dis..

[20] Harms A.S., Ferreira S.A., Romero-Ramos M. (2021). Periphery and brain, innate and adaptive immunity in Parkinson’s disease.. Acta Neuropathol..

[21] Yi M., Li J., Jian S., Li B., Huang Z., Shu L., Zhang Y. (2023). Quantitative and causal analysis for inflammatory genes and the risk of Parkinson’s disease.. Front. Immunol..

[22] Abbott N.J., Patabendige A.A.K., Dolman D.E.M., Yusof S.R., Begley D.J. (2010). Structure and function of the blood–brain barrier.. Neurobiol. Dis..

[23] Labzin L.I., Heneka M.T., Latz E. (2018). Innate immunity and neurodegeneration.. Annu. Rev. Med..

[24] Huang X., Hussain B., Chang J. (2021). Peripheral inflammation and blood–brain barrier disruption: Effects and mechanisms.. CNS Neurosci. Ther..

[25] Wilhelm I., Nyúl-Tóth Á., Suciu M., Hermenean A., Krizbai I.A. (2016). Heterogeneity of the blood-brain barrier.. Tissue Barriers.

[26] Mayne K., White J.A., McMurran C.E., Rivera F.J., de la Fuente A.G. (2020). Aging and neurodegenerative disease: Is the adaptive immune system a friend or foe?. Front. Aging Neurosci..

[27] Glass C.K., Saijo K., Winner B., Marchetto M.C., Gage F.H. (2010). Mechanisms underlying inflammation in neurodegeneration.. Cell.

[28] Stephenson J., Nutma E., van der Valk P., Amor S. (2018). Inflammation in CNS neurodegenerative diseases.. Immunology.

[29] Fathi M., Vakili K., Yaghoobpoor S., Qadirifard M.S., Kosari M., Naghsh N.; (2022). Asgari taei, A.; Klegeris, A.; Dehghani, M.; Bahrami, A.; Taheri, H.; Mohamadkhani, A.; Hajibeygi, R.; Rezaei Tavirani, M.; Sayehmiri, F. Pre-clinical studies identifying molecular pathways of neuroinflammation in Parkinson’s disease: A systematic review.. Front. Aging Neurosci..

[30] Gorecki A.M., Anyaegbu C.C., Anderton R.S. (2021). TLR2 and TLR4 in Parkinson’s disease pathogenesis: The environment takes a toll on the gut.. Transl. Neurodegener..

[31] Bellucci A., Bubacco L., Longhena F., Parrella E., Faustini G., Porrini V., Bono F., Missale C., Pizzi M. (2020). Nuclear Factor-κB dysregulation and α-synuclein pathology: Critical interplay in the pathogenesis of Parkinson’s disease.. Front. Aging Neurosci..

[32] Juranek J., Mukherjee K., Kordas B.; (2022). Załęcki, M.; Korytko, A.; Zglejc-Waszak, K.; Szuszkiewicz, J.; Banach, M. Role of RAGE in the pathogenesis of neurological disorders.. Neurosci. Bull..

[33] Spulber S., Bartfai T., Schultzberg M. (2009). IL-1/IL-1ra balance in the brain revisited: Evidence from transgenic mouse models.. Brain Behav. Immun..

[34] Bai H., Zhang Q. (2021). Activation of NLRP3 inflammasome and onset of Alzheimer’s disease.. Front. Immunol..

[35] Martin-Ruiz C., Williams-Gray C.H., Yarnall A.J., Boucher J.J., Lawson R.A., Wijeyekoon R.S., Barker R.A., Kolenda C., Parker C., Burn D.J., Von Zglinicki T., Saretzki G. (2020). Senescence and inflammatory markers for predicting clinical progression in Parkinson’s disease: The ICICLE-PD Study.. J. Parkinsons Dis..

[36] Lara P.C., Macías-Verde D., Burgos-Burgos J. (2020). Age-induced NLRP3 inflammasome over-activation increases lethality of SARS-CoV-2 pneumonia in elderly patients.. Aging Dis..

[37] Stout-Delgado H.W., Vaughan S.E., Shirali A.C., Jaramillo R.J., Harrod K.S. (2012). Impaired NLRP3 inflammasome function in elderly mice during influenza infection is rescued by treatment with nigericin.. J. Immunol..

[38] Nagatsu T., Mogi M., Ichinose H., Togari A. (2000). Changes in cytokines and neurotrophins in Parkinson’s disease.. J. Neural Transm. Suppl..

[39] Zhang P., Shao X.Y., Qi G.J., Chen Q., Bu L.L., Chen L.J., Shi J., Ming J., Tian B. (2016). Cdk5-dependent activation of neuronal inflammasomes in Parkinson’s disease.. Mov. Disord..

[40] Kitazawa M., Cheng D., Tsukamoto M.R., Koike M.A., Wes P.D., Vasilevko V., Cribbs D.H., LaFerla F.M. (2011). Blocking IL-1 signaling rescues cognition, attenuates tau pathology, and restores neuronal β-catenin pathway function in an Alzheimer’s disease model.. J. Immunol..

[41] Wang W., Nguyen L.T.T., Burlak C., Chegini F., Guo F., Chataway T., Ju S., Fisher O.S., Miller D.W., Datta D., Wu F., Wu C.X., Landeru A., Wells J.A., Cookson M.R., Boxer M.B., Thomas C.J., Gai W.P., Ringe D., Petsko G.A., Hoang Q.Q. (2016). Caspase-1 causes truncation and aggregation of the Parkinson’s disease-associated protein α-synuclein.. Proc. Natl. Acad. Sci..

[42] Hurelbrink C.B., Armstrong R.J.E., Luheshi L.M., Dunnett S.B., Rosser A.E., Barker R.A. (2001). Death of dopaminergic neurons in vitro and in nigral grafts: Reevaluating the role of caspase activation.. Exp. Neurol..

[43] Caputi V., Giron M. (2018). Microbiome-gut-brain axis and toll-like receptors in Parkinson’s disease.. Int. J. Mol. Sci..

[44] Howe A.M., Burke S., O’Reilly M.E., McGillicuddy F.C., Costello D.A. (2022). Palmitic acid and oleic acid differently modulate tlr2-mediated inflammatory responses in microglia and macrophages.. Mol. Neurobiol..

[45] Minoretti P., Gazzaruso C., Vito C.D., Emanuele E., Bianchi M., Coen E., Reino M., Geroldi D. (2006). Effect of the functional toll-like receptor 4 Asp299Gly polymorphism on susceptibility to late-onset Alzheimer’s disease.. Neurosci. Lett..

[46] Okun E., Griffioen K.J., Lathia J.D., Tang S.C., Mattson M.P., Arumugam T.V. (2009). Toll-like receptors in neurodegeneration.. Brain Res. Brain Res. Rev..

[47] Liddelow S.A., Barres B.A. (2017). Reactive Astrocytes: Production, Function, and Therapeutic Potential.. Immunity.

[48] Labib D., Wang Z., Prakash P., Zimmer M., Smith M.D., Frazel P.W., Barbar L., Sapar M.L., Calabresi P.A., Peng J., Liddelow S.A., Fossati V. (2022). Proteomic Alterations and Novel Markers of Neurotoxic Reactive Astrocytes in Human Induced Pluripotent Stem Cell Models.. Front. Mol. Neurosci..

[49] Zhao Y., Bhattacharjee S., Jones B.M., Hill J., Dua P., Lukiw W.J. (2014). Regulation of neurotropic signaling by the inducible, NF-kB-sensitive miRNA-125b in Alzheimer’s disease (AD) and in primary human neuronal-glial (HNG) cells.. Mol. Neurobiol..

[50] Singh S., Singh T.G. (2020). Role of Nuclear Factor Kappa B (NF-κB) signalling in neurodegenerative diseases: A mechanistic approach.. Curr. Neuropharmacol..

[51] Dou F., Chu X., Zhang B., Liang L., Lu G., Ding J., Chen S. (2018). EriB targeted inhibition of microglia activity attenuates MPP+ induced DA neuron injury through the NF-κB signaling pathway.. Mol. Brain.

[52] Rauf A., Badoni H., Abu-Izneid T., Olatunde A., Rahman M.M., Painuli S., Semwal P., Wilairatana P., Mubarak M.S. (2022). Neuroinflammatory markers: Key indicators in the pathology of neurodegenerative diseases.. Molecules.

[53] Huang Y., Erdmann N., Peng H., Zhao Y., Zheng J. (2005). The role of TNF related apoptosis-inducing ligand in neurodegenerative diseases.. Cell. Mol. Immunol..

[54] Uberti D., Cantarella G., Facchetti F., Cafici A., Grasso G., Bernardini R., Memo M. (2004). TRAIL is expressed in the brain cells of Alzheimer’s disease patients.. Neuroreport.

[55] Akiyama H., Barger S., Barnum S., Bradt B., Bauer J., Cole G.M., Cooper N.R., Eikelenboom P., Emmerling M., Fiebich B.L., Finch C.E., Frautschy S., Griffin W.S., Hampel H., Hull M., Landreth G., Lue L., Mrak R., Mackenzie I.R., McGeer P.L., O’Banion M.K., Pachter J., Pasinetti G., Plata-Salaman C., Rogers J., Rydel R., Shen Y., Streit W., Strohmeyer R., Tooyoma I., Van Muiswinkel F.L., Veerhuis R., Walker D., Webster S., Wegrzyniak B., Wenk G., Wyss-Coray T. (2000). Inflammation and Alzheimer’s disease.. Neurobiol. Aging.

[56] Tarkowski E., Liljeroth A.M., Nilsson Å., Minthon L., Blennow K. (2001). Decreased levels of intrathecal interleukin 1 receptor antagonist in Alzheimer’s disease.. Dement. Geriatr. Cogn. Disord..

[57] He P., Zhong Z., Lindholm K., Berning L., Lee W., Lemere C., Staufenbiel M., Li R., Shen Y. (2007). Deletion of tumor necrosis factor death receptor inhibits amyloid β generation and prevents learning and memory deficits in Alzheimer’s mice.. J. Cell Biol..

[58] Hickman S.E., Allison E.K., El Khoury J. (2008). Microglial dysfunction and defective beta-amyloid clearance pathways in aging Alzheimer’s disease mice.. J. Neurosci..

[59] Nutma E., van Gent D., Amor S., Peferoen L.A.N. (2020). Astrocyte and oligodendrocyte cross-talk in the central nervous system.. Cells.

[60] Santoro A., Spinelli C.C., Martucciello S., Nori S.L., Capunzo M., Puca A.A., Ciaglia E. (2018). Innate immunity and cellular senescence: The good and the bad in the developmental and aged brain.. J. Leukoc. Biol..

[61] Tan Z.S., Beiser A.S., Vasan R.S., Roubenoff R., Dinarello C.A., Harris T.B., Benjamin E.J., Au R., Kiel D.P., Wolf P.A., Seshadri S. (2007). Inflammatory markers and the risk of Alzheimer disease: The Framingham Study.. Neurology.

[62] Burré J., Sharma M., Südhof T.C. (2018). Cell biology and pathophysiology of α-synuclein.. Cold Spring Harb. Perspect. Med..

[63] Nakanishi H. (2020). Microglial cathepsin B as a key driver of inflammatory brain diseases and brain aging.. Neural Regen. Res..

[64] Kim C., Ho D.H., Suk J.E., You S., Michael S., Kang J., Joong Lee S., Masliah E., Hwang D., Lee H.J., Lee S.J. (2013). Neuron-released oligomeric α-synuclein is an endogenous agonist of TLR2 for paracrine activation of microglia.. Nat. Commun..

[65] Xie Y.X., Naseri N.N., Fels J., Kharel P., Na Y., Lane D., Burré J., Sharma M. (2022). Lysosomal exocytosis releases pathogenic α-synuclein species from neurons in synucleinopathy models.. Nat. Commun..

[66] Lashuel H.A., Overk C.R., Oueslati A., Masliah E. (2013). The many faces of α-synuclein: from structure and toxicity to therapeutic target.. Nat. Rev. Neurosci..

[67] Bendor J.T., Logan T.P., Edwards R.H. (2013). The function of α-synuclein.. Neuron.

[68] Soraci L., Gambuzza M.E., Biscetti L., Laganà P., Lo Russo C., Buda A., Barresi G., Corsonello A., Lattanzio F., Lorello G., Filippelli G., Marino S. (2023). Toll-like receptors and NLRP3 inflammasome-dependent pathways in Parkinson’s disease: Mechanisms and therapeutic implications.. J. Neurol..

[69] Volpicelli-Daley L., Brundin P. (2018). Prion-like propagation of pathology in Parkinson disease.. Handb. Clin. Neurol..

[70] Noguchi-Shinohara M., Ono K. (2023). The mechanisms of the roles of α-synuclein, amyloid-β, and tau protein in the lewy body diseases: pathogenesis, early detection, and therapeutics.. Int. J. Mol. Sci..

[71] Schrag A. (2004). Psychiatric aspects of Parkinson’s disease.. J. Neurol..

[72] Subramanian A., Tamilanban T., Alsayari A., Ramachawolran G., Wong L.S., Sekar M., Gan S.H., Subramaniyan V., Chinni S.V., Izzati Mat Rani N.N., Suryadevara N., Wahab S. (2022). Trilateral association of autophagy, mTOR and Alzheimer’s disease: Potential pathway in the development for Alzheimer’s disease therapy.. Front. Pharmacol..

[73] Kostiuchenko O., Lushnikova I., Kowalczyk M., Skibo G. (2022). mTOR/α-ketoglutarate-mediated signaling pathways in the context of brain neurodegeneration and neuroprotection.. BBA Adv..

[74] Blagov A.V., Grechko A.V., Nikiforov N.G., Borisov E.E., Sadykhov N.K., Orekhov A.N. (2022). Role of impaired mitochondrial dynamics processes in the pathogenesis of Alzheimer’s disease.. Int. J. Mol. Sci..

[75] Ikeda-Matsuo Y., Miyata H., Mizoguchi T., Ohama E., Naito Y., Uematsu S., Akira S., Sasaki Y., Tanabe M. (2019). Microsomal prostaglandin E synthase-1 is a critical factor in dopaminergic neurodegeneration in Parkinson’s disease.. Neurobiol. Dis..

[76] Mi Y., Qi G., Vitali F., Shang Y., Raikes A.C., Wang T., Jin Y., Brinton R.D., Gu H., Yin F. (2023). Loss of fatty acid degradation by astrocytic mitochondria triggers neuroinflammation and neurodegeneration.. Nat. Metab..

[77] Kulminski A.M., Jain-Washburn E., Loiko E., Loika Y., Feng F., Culminskaya I. (2022). Associations of the APOE ε2 and ε4 alleles and polygenic profiles comprising APOE-TOMM40-APOC1 variants with Alzheimer’s disease biomarkers.. Aging.

[78] Mu G., Ren C., Zhang Y., Lu B., Feng J., Wu D., Xu X., Ou C. (2023). Amelioration of central neurodegeneration by docosahexaenoic acid in trigeminal neuralgia rats through the regulation of central neuroinflammation.. Int. Immunopharmacol..

[79] Xie A., Ensink E., Li P.; (2022). Gordevičius, J.; Marshall, L.L.; George, S.; Pospisilik, J.A.; Aho, V.T.E.; Houser, M.C.; Pereira, P.A.B.; Rudi, K.; Paulin, L.; Tansey, M.G.; Auvinen, P.; Brundin, P.; Brundin, L.; Labrie, V.; Scheperjans, F. Bacterial butyrate in parkinson’s disease is linked to epigenetic changes and depressive symptoms.. Mov. Disord..

[80] Verhaar B.J.H., Hendriksen H.M.A., de Leeuw F.A., Doorduijn A.S., van Leeuwenstijn M., Teunissen C.E., Barkhof F., Scheltens P., Kraaij R., van Duijn C.M., Nieuwdorp M., Muller M., van der Flier W.M. (2022). Gut microbiota composition is related to ad pathology.. Front. Immunol..

[81] Cammann D., Lu Y., Cummings M.J., Zhang M.L., Cue J.M., Do J., Ebersole J., Chen X., Oh E.C., Cummings J.L., Chen J. (2023). Genetic correlations between Alzheimer’s disease and gut microbiome genera.. Sci. Rep..

[82] Lang Y., Chu F., Shen D., Zhang W., Zheng C., Zhu J., Cui L. (2018). Role of inflammasomes in neuroimmune and neurodegenerative diseases: A systematic review.. Mediators Inflamm..

[83] Miao J., Ma H., Yang Y., Liao Y., Lin C., Zheng J., Yu M., Lan J. (2023). Microglia in Alzheimer’s disease: Pathogenesis, mechanisms, and therapeutic potentials.. Front. Aging Neurosci..

[84] Wes P.D., Holtman I.R., Boddeke E.W.G.M., Möller T., Eggen B.J.L. (2016). Next generation transcriptomics and genomics elucidate biological complexity of microglia in health and disease.. Glia.

[85] Holtman I.R., Raj D.D., Miller J.A., Schaafsma W., Yin Z., Brouwer N., Wes P.D., Möller T., Orre M., Kamphuis W., Hol E.M., Boddeke E.W.G.M., Eggen B.J.L. (2015). Induction of a common microglia gene expression signature by aging and neurodegenerative conditions: A co-expression meta-analysis.. Acta Neuropathol. Commun..

[86] Pan J., Ma N., Yu B., Zhang W., Wan J. (2020). Transcriptomic profiling of microglia and astrocytes throughout aging.. J. Neuroinflammation.

[87] Spurrier J., Nicholson L., Fang X.T., Stoner A.J., Toyonaga T., Holden D., Siegert T.R., Laird W., Allnutt M.A., Chiasseu M., Brody A.H., Takahashi H., Nies S.H., Cañamás A.P., Sadasivam P., Lee S., Li S., Zhang L., Huang Y.H., Carson R.E., Cai Z., Strittmatter S.M. (2022). Reversal of synapse loss in Alzheimer mouse models by targeting mGluR5 to prevent synaptic tagging by C1Q.. Sci. Transl. Med..

[88] Balog B.M., Sonti A., Zigmond R.E. (2023). Neutrophil biology in injuries and diseases of the central and peripheral nervous systems.. Prog. Neurobiol..

[89] Aries M.L., Hensley-McBain T. (2023). Neutrophils as a potential therapeutic target in Alzheimer’s disease.. Front. Immunol..

[90] Harcha P.A., Garcés P., Arredondo C., Fernández G., Sáez J.C., van Zundert B. (2021). Mast cell and astrocyte hemichannels and their role in alzheimer’s disease, ALS, and harmful stress conditions.. Int. J. Mol. Sci..

[91] Wang S., van de Pavert S.A. (2022). Innate lymphoid cells in the central nervous system.. Front. Immunol..

[92] Brauning A., Rae M., Zhu G., Fulton E., Admasu T.D., Stolzing A., Sharma A. (2022). Aging of the immune system: Focus on natural killer cells phenotype and functions.. Cells.

[93] Prager I., Watzl C. (2019). Mechanisms of natural killer cell-mediated cellular cytotoxicity.. J. Leukoc. Biol..

[94] Menees K.B., Lee J.K. (2022). New insights and implications of natural killer cells in parkinson’s disease.. J. Parkinsons Dis..

[95] Zhang L., Zhang Y., Fan D. (2022). Pathological role of natural killer cells in parkinson’s disease: A systematic review.. Front. Aging Neurosci..

[96] Muñiz-Castrillo S., Vogrig A., Honnorat J. (2020). Associations between HLA and autoimmune neurological diseases with autoantibodies.. Auto Immun. Highlights.

[97] Boon B.D.C., Hoozemans J.J.M., Lopuhaä B., Eigenhuis K.N., Scheltens P., Kamphorst W., Rozemuller A.J.M., Bouwman F.H. (2018). Neuroinflammation is increased in the parietal cortex of atypical Alzheimer’s disease.. J. Neuroinflammation.

[98] Wang Z.T., Chen S.D., Xu W., Chen K.L., Wang H.F., Tan C.C., Cui M., Dong Q., Tan L., Yu J.T. (2019). Genome-wide association study identifies CD1A associated with rate of increase in plasma neurofilament light in non-demented elders.. Aging.

[99] Chew H., Solomon V.A., Fonteh A.N. (2020). Involvement of lipids in Alzheimer’s disease pathology and potential therapies.. Front. Physiol..

[100] Al-kuraishy H.M., Al-Gareeb A.I., Alexiou A., Papadakis M., Alsayegh A.A., Almohmadi N.H., Saad H.M., Batiha G.E.S. (2023). Pros and cons for statins use and risk of Parkinson’s disease: An updated perspective.. Pharmacol. Res. Perspect..

[101] Sulzer D., Alcalay R.N., Garretti F., Cote L., Kanter E., Agin-Liebes J., Liong C., McMurtrey C., Hildebrand W.H., Mao X., Dawson V.L., Dawson T.M., Oseroff C., Pham J., Sidney J., Dillon M.B., Carpenter C., Weiskopf D., Phillips E., Mallal S., Peters B., Frazier A., Lindestam A.C.S., Sette A. (2017). T cells from patients with Parkinson’s disease recognize α-synuclein peptides.. Nature.

[102] Williams G.P., Schonhoff A.M., Jurkuvenaite A., Gallups N.J., Standaert D.G., Harms A.S. (2021). CD4 T cells mediate brain inflammation and neurodegeneration in a mouse model of Parkinson’s disease.. Brain.

[103] Iba M., Kim C., Sallin M., Kwon S., Verma A., Overk C., Rissman R.A., Sen R., Sen J.M., Masliah E. (2020). Neuroinflammation is associated with infiltration of T cells in Lewy body disease and α-synuclein transgenic models.. J. Neuroinflammation.

[104] Lyman M., Lloyd D.G., Ji X., Vizcaychipi M.P., Ma D. (2014). Neuroinflammation: The role and consequences.. Neurosci. Res..

[105] Carrasco E., Gómez de las Heras M.M., Gabandé-Rodríguez E., Desdín-Micó G., Aranda J.F., Mittelbrunn M. (2022). The role of T cells in age-related diseases.. Nat. Rev. Immunol..

[106] Gate D., Saligrama N., Leventhal O., Yang A.C., Unger M.S., Middeldorp J., Chen K., Lehallier B., Channappa D., De Los Santos M.B., McBride A., Pluvinage J., Elahi F., Tam G.K.Y., Kim Y., Greicius M., Wagner A.D., Aigner L., Galasko D.R., Davis M.M., Wyss-Coray T. (2020). Clonally expanded CD8 T cells patrol the cerebrospinal fluid in Alzheimer’s disease.. Nature.

[107] Mietelska-Porowska A., Wojda U. (2017). T lymphocytes and inflammatory mediators in the interplay between brain and blood in Alzheimer’s disease: Potential pools of new biomarkers.. J. Immunol. Res..

[108] Rezai-Zadeh K., Gate D., Town T. (2009). CNS infiltration of peripheral immune cells: D-Day for neurodegenerative disease?. J. Neuroimmune Pharmacol..

[109] Dai L., Shen Y. (2021). Insights into Tcell dysfunction in Alzheimer’s disease.. Aging Cell.

[110] Machhi J., Yeapuri P., Lu Y., Foster E., Chikhale R., Herskovitz J., Namminga K.L., Olson K.E., Abdelmoaty M.M., Gao J., Quadros R.M., Kiyota T., Jingjing L., Kevadiya B.D., Wang X., Liu Y., Poluektova L.Y., Gurumurthy C.B., Mosley R.L., Gendelman H.E. (2021). CD4+ effector T cells accelerate Alzheimer’s disease in mice.. J. Neuroinflammation.

[111] Monsonego A., Zota V., Karni A., Krieger J.I., Bar-Or A., Bitan G., Budson A.E., Sperling R., Selkoe D.J., Weiner H.L. (2003). Increased T cell reactivity to amyloid β protein in older humans and patients with Alzheimer disease.. J. Clin. Invest..

[112] Kustrimovic N., Comi C., Magistrelli L., Rasini E., Legnaro M., Bombelli R., Aleksic I., Blandini F., Minafra B., Riboldazzi G., Sturchio A., Mauri M., Bono G., Marino F., Cosentino M. (2018). Parkinson’s disease patients have a complex phenotypic and functional Th1 bias: Cross-sectional studies of CD4+ Th1/Th2/T17 and Treg in drug-naïve and drug-treated patients.. J. Neuroinflammation.

[113] Saunders J.A.H., Estes K.A., Kosloski L.M., Allen H.E., Dempsey K.M., Torres-Russotto D.R., Meza J.L., Santamaria P.M., Bertoni J.M., Murman D.L., Ali H.H., Standaert D.G., Mosley R.L., Gendelman H.E. (2012). CD4+ regulatory and effector/memory T cell subsets profile motor dysfunction in Parkinson’s disease.. J. Neuroimmune Pharmacol..

[114] Xu Y., Li Y., Wang C., Han T., Liu H., Sun L., Hong J., Hashimoto M., Wei J. (2023). The reciprocal interactions between microglia and T cells in Parkinson’s disease: A double-edged sword.. J. Neuroinflammation.

[115] Vacinova G., Vejražkova D., Rusina R., Holmerová I.; (2021). Vaňková, H.; Jarolímová, E.; Včelák, J.; Bendlová, B.; Vaňková, M. Regulated upon activation, normal T cell expressed and secreted (RANTES) levels in the peripheral blood of patients with Alzheimer’s disease.. Neural Regen. Res..

[116] Schwartz M., Baruch K. (2014). Breaking peripheral immune tolerance to CNS antigens in neurodegenerative diseases: Boosting autoimmunity to fight-off chronic neuroinflammation.. J. Autoimmun..

[117] Chen X., Firulyova M., Manis M., Herz J., Smirnov I., Aladyeva E., Wang C., Bao X., Finn M.B., Hu H., Shchukina I., Kim M.W., Yuede C.M., Kipnis J., Artyomov M.N., Ulrich J.D., Holtzman D.M. (2023). Microglia-mediated T cell infiltration drives neurodegeneration in tauopathy.. Nature.

[118] Subbarayan M.S., Hudson C., Moss L.D., Nash K.R., Bickford P.C. (2020). T cell infiltration and upregulation of MHCII in microglia leads to accelerated neuronal loss in an α-synuclein rat model of Parkinson’s disease.. J. Neuroinflammation.

[119] Cai H.Y., Fu X.X., Jiang H., Han S. (2021). Adjusting vascular permeability, leukocyte infiltration, and microglial cell activation to rescue dopaminergic neurons in rodent models of Parkinson’s disease.. NPJ Parkinsons Dis..

[120] Liu Y., Sorce S., Nuvolone M., Domange J., Aguzzi A. (2018). Lymphocyte activation gene 3 (Lag3) expression is increased in prion infections but does not modify disease progression.. Sci. Rep..

[121] Guo W., Zhou M., Qiu J., Lin Y., Chen X., Huang S., Mo M., Liu H., Peng G., Zhu X., Xu P. (2019). Association of LAG3 genetic variation with an increased risk of PD in Chinese female population.. J. Neuroinflammation.

[122] García-Martín E., Pastor P., Gómez-Tabales J., Alonso-Navarro H., Alvarez I., Buongiorno M., Cerezo-Arias M.O., Aguilar M., Agúndez J.A.G., Jiménez-Jiménez F.J. (2022). Association between LAG3/CD4 gene variants and risk of Parkinson’s disease.. Eur. J. Clin. Invest..

[123] Cui S., Du J.J., Liu S.H., Meng J., Lin Y.Q., Li G., He Y.X., Zhang P.C., Chen S., Wang G. (2019). Serum soluble lymphocyte activation gene3 as a diagnostic biomarker in Parkinson’s disease: A pilot multicenter study.. Mov. Disord..

[124] Roy A., Choudhury S., Banerjee R., Basu P., Kumar H. (2021). Soluble LAG-3 and Toll-interacting protein: Novel upstream neuro-inflammatory markers in Parkinson’s disease.. Parkinsonism Relat. Disord..

[125] Saresella M., Calabrese E., Marventano I., Piancone F., Gatti A., Calvo M.G., Nemni R., Clerici M. (2010). PD1 negative and PD1 positive CD4+ T regulatory cells in mild cognitive impairment and Alzheimer’s disease.. J. Alzheimers Dis..

[126] Olson K.E., Mosley R.L., Gendelman H.E. (2022). The potential for treg-enhancing therapies in nervous system pathologies.. Clin. Exp. Immunol..

[127] Beers D.R., Zhao W., Wang J., Zhang X., Wen S., Neal D., Thonhoff J.R., Alsuliman A.S., Shpall E.J., Rezvani K., Appel S.H. (2017). ALS patients’ regulatory T lymphocytes are dysfunctional, and correlate with disease progression rate and severity.. JCI Insight.

[128] Schröder J.B., Pawlowski M., Meyer zu Hörste G., Gross C.C., Wiendl H., Meuth S.G., Ruck T., Warnecke T. (2018). Immune cell activation in the cerebrospinal fluid of patients with Parkinson’s disease.. Front. Neurol..

[129] Stym-Popper G., Matta K., Chaigneau T., Rupra R., Demetriou A., Fouquet S., Dansokho C., Toly-Ndour C., Dorothée G. (2023). Regulatory T cells decrease C3-positive reactive astrocytes in Alzheimer-like pathology.. J. Neuroinflammation.

[130] Ciccocioppo F., Lanuti P., Pierdomenico L., Simeone P., Bologna G., Ercolino E., Buttari F., Fantozzi R., Thomas A., Onofrj M., Centonze D., Miscia S., Marchisio M. (2019). The characterization of regulatory t-cell profiles in Alzheimer’s disease and multiple sclerosis.. Sci. Rep..

[131] Baruch K., Rosenzweig N., Kertser A., Deczkowska A., Sharif A.M., Spinrad A., Tsitsou-Kampeli A., Sarel A., Cahalon L., Schwartz M. (2015). Breaking immune tolerance by targeting Foxp3+ regulatory T cells mitigates Alzheimer’s disease pathology.. Nat. Commun..

[132] Novakova Martinkova J., Ferretti M.T., Ferrari A., Lerch O., Matuskova V., Secnik J., Hort J. (2023). Longitudinal progression of choroid plexus enlargement is associated with female sex, cognitive decline and ApoE E4 homozygote status.. Front. Psychiatry.

[133] Yang H., Park S.Y., Baek H., Lee C., Chung G., Liu X., Lee J.H., Kim B., Kwon M., Choi H., Kim H.J., Kim J.Y., Kim Y., Lee Y.S., Lee G., Kim S.K., Kim J.S., Chang Y.T., Jung W.S., Kim K.H., Bae H. (2022). Adoptive therapy with amyloid-β specific regulatory T cells alleviates Alzheimer’s disease.. Theranostics.

[134] Moore J.R., Hubler S.L., Nelson C.D., Nashold F.E., Spanier J.A., Hayes C.E. (2018). 1,25-Dihydroxyvitamin D3 increases the methionine cycle, CD4+ T cell DNA methylation and Helios+Foxp3+ T regulatory cells to reverse autoimmune neurodegenerative disease.. J. Neuroimmunol..

[135] Janjusevic M., Gagno G., Fluca A.L., Padoan L., Beltrami A.P., Sinagra G., Moretti R., Aleksova A. (2022). The peculiar role of vitamin D in the pathophysiology of cardiovascular and neurodegenerative diseases.. Life Sci..

[136] Shi Y., Wei B., Li L., Wang B., Sun M. (2022). Th17 cells and inflammation in neurological disorders: Possible mechanisms of action.. Front. Immunol..

[137] Sommer A., Marxreiter F., Krach F., Fadler T., Grosch J., Maroni M., Graef D., Eberhardt E., Riemenschneider M.J., Yeo G.W., Kohl Z., Xiang W., Gage F.H., Winkler J., Prots I., Winner B. (2019). Th17 lymphocytes induce neuronal cell death in a human iPSC-based model of Parkinson’s disease.. Cell Stem Cell.

[138] Li J. (2023). Zhao, J.; Chen, L.; Gao, H.; Zhang, J.; Wang, D.; Zou, Y.; Qin, Q.; Qu, Y.; Li, J.; Xiong, Y.; Min, Z.; Yan, M.; Mao, Z.; Xue, Z. α-Synuclein induces Th17 differentiation and impairs the function and stability of Tregs by promoting RORC transcription in Parkinson’s disease.. Brain Behav. Immun..

[139] Mohammadi S., (2018). V.; Ravari, A.; Mirzaei, T.; Zare-Bidaki, M.; Asadikaram, G.; Arababadi, M.K. IL-17A and IL-23: Plausible risk factors to induce age-associated inflammation in Alzheimer’s disease.. Immunol. Invest..

[140] Biragyn A., Aliseychik M., Rogaev E. (2017). Potential importance of B cells in aging and aging-associated neurodegenerative diseases.. Semin. Immunopathol..

[141] Sabatino J.J., Pröbstel A.K., Zamvil S.S. (2019). B cells in autoimmune and neurodegenerative central nervous system diseases.. Nat. Rev. Neurosci..

[142] Orr C.F., Rowe D.B., Mizuno Y., Mori H., Halliday G.M. (2005). A possible role for humoral immunity in the pathogenesis of Parkinson’s disease.. Brain.

[143] Du Y., Dodel R., Hampel H., Buerger K., Lin S., Eastwood B., Bales K., Gao F., Moeller H.J., Oertel W., Farlow M., Paul S. (2001). Reduced levels of amyloid -peptide antibody in Alzheimer disease.. Neurology.

[144] Hyman B.T., Smith C., Buldyrev I., Whelan C., Brown H., Tang M.X., Mayeux R. (2001). Autoantibodies to amyloid-? and Alzheimer’s disease.. Ann. Neurol..

[145] Weksler M.E., Relkin N., Turkenich R., LaRusse S., Zhou L., Szabo P. (2002). Patients with Alzheimer disease have lower levels of serum anti-amyloid peptide antibodies than healthy elderly individuals.. Exp. Gerontol..

[146] DeMarshall C.A., Viviano J., Emrani S., Thayasivam U., Godsey G.A., Sarkar A., Belinka B., Libon D.J., Nagele R.G. (2023). Early detection of alzheimer’s disease-related pathology using a multi-disease diagnostic platform employing autoantibodies as blood-based biomarkers.. J. Alzheimers Dis..

[147] Carvey P.M., McRae A., Lint T.F., Ptak L.R., Lo E.S., Goetz C.G., Klawans H.L. (1991). The potential use of a dopamine neuron antibody and a striatal-derived neurotrophic factor as diagnostic markers in Parkinson’s disease..

[148] Chen S., Le W.D., Xie W.J., Alexianu M.E., Engelhardt J.I., Siklós L., Appel S.H. (1998). Experimental destruction of substantia nigra initiated by Parkinson disease immunoglobulins.. Arch. Neurol..

[149] Le W.D., Rowe D.B., Jankovic J., Xie W., Appel S.H. (1999). Effects of cerebrospinal fluid from patients with Parkinson disease on dopaminergic cells.. Arch. Neurol..

[150] Papachroni K.K., Ninkina N., Papapanagiotou A., Hadjigeorgiou G.M., Xiromerisiou G., Papadimitriou A., Kalofoutis A., Buchman V.L. (2007). Autoantibodies to alpha-synuclein in inherited Parkinson’s disease.. J. Neurochem..

[151] Shalash A., Salama M., Makar M., Roushdy T., Elrassas H.H., Mohamed W., El-Balkimy M., Abou D.M. (2017). Elevated serum α-synuclein autoantibodies in patients with Parkinson’s disease relative to Alzheimer’s disease and controls.. Front. Neurol..

[152] Besong-Agbo D., Wolf E., Jessen F., Oechsner M., Hametner E., Poewe W., Reindl M., Oertel W.H., Noelker C., Bacher M., Dodel R. (2013). Naturally occurring -synuclein autoantibody levels are lower in patients with Parkinson disease.. Neurology.

[153] Horvath I., Iashchishyn I.A., Forsgren L., Morozova-Roche L.A. (2017). Immunochemical detection of α-synuclein autoantibodies in Parkinson’s disease: Correlation between plasma and cerebrospinal fluid levels.. ACS Chem. Neurosci..

[154] Akhtar R.S., Licata J.P., Luk K.C., Shaw L.M., Trojanowski J.Q., Lee V.M.Y. (2018). Measurements of auto-antibodies to α-synuclein in the serum and cerebral spinal fluids of patients with Parkinson’s disease.. J. Neurochem..

[155] Double K.L., Rowe D.B., Carew-Jones F.M., Hayes M., Chan D.K.Y., Blackie J., Corbett A., Joffe R., Fung V.S., Morris J., Riederer P., Gerlach M., Halliday G.M. (2009). Anti-melanin antibodies are increased in sera in Parkinson’s disease.. Exp. Neurol..

[156] Zappia M., Crescibene L., Bosco D., Arabia G., Nicoletti G., Bagalà A., Bastone L., Napoli I.D., Caracciolo M., Bonavita S., Di Costanzo A., Gambardella A., Quattrone A. (2002). Anti-GM1 ganglioside antibodies in Parkinson’s disease.. Acta Neurol. Scand..

[157] De Virgilio A., Greco A., Fabbrini G., Inghilleri M., Rizzo M.I., Gallo A., Conte M., Rosato C., Ciniglio Appiani M., de Vincentiis M. (2016). Parkinson’s disease: Autoimmunity and neuroinflammation.. Autoimmun. Rev..

[158] Benkler M., Agmon-Levin N., Hassin-Baer S., Cohen O.S., Ortega-Hernandez O.D., Levy A., Moscavitch S.D., Szyper-Kravitz M., Damianovich M., Blank M., Chapman J., Shoenfeld Y. (2012). Immunology, autoimmunity, and autoantibodies in Parkinson’s disease.. Clin. Rev. Allergy Immunol..

[159] (2017). Papuć, E.; Rejdak, K. Anti-MAG autoantibodies are increased in Parkinson’s disease but not in atypical parkinsonism.. J. Neural Transm..

[160] Honorat J.A., McKeon A. (2017). Autoimmune movement disorders: A clinical and laboratory approach.. Curr. Neurol. Neurosci. Rep..

[161] Caggiu E., Paulus K., Arru G., Piredda R., Sechi G.P., Sechi L.A. (2016). Humoral cross reactivity between α-synuclein and herpes simplex-1 epitope in Parkinson’s disease, a triggering role in the disease?. J. Neuroimmunol..

[162] Cebrián C., Zucca F.A., Mauri P., Steinbeck J.A., Studer L., Scherzer C.R., Kanter E., Budhu S., Mandelbaum J., Vonsattel J.P., Zecca L., Loike J.D., Sulzer D. (2014). MHC-I expression renders catecholaminergic neurons susceptible to T-cell-mediated degeneration.. Nat. Commun..

[163] Jiang T., Li G., Xu J., Gao S., Chen X. (2018). The challenge of the pathogenesis of parkinson’s disease: Is autoimmunity the culprit?. Front. Immunol..

[164] Oberländer U., Pletinckx K., Döhler A., Müller N., Lutz M.B., Arzberger T., Riederer P., Gerlach M., Koutsilieri E., Scheller C. (2011). Neuromelanin is an immune stimulator for dendritic cells in vitro.. BMC Neurosci..

[165] Koutsilieri E., Lutz M.B., Scheller C. (2013). Autoimmunity, dendritic cells and relevance for Parkinson’s disease.. J. Neural Transm..

[166] Depboylu C., Schäfer M.K.H., Arias-Carrión O., Oertel W.H., Weihe E., Höglinger G.U. (2011). Possible involvement of complement factor C1q in the clearance of extracellular neuromelanin from the substantia nigra in Parkinson disease.. J. Neuropathol. Exp. Neurol..

[167] Alberici A., Cristillo V., Gazzina S., Benussi A., Padovani A., Borroni B. (2018). Autoimmunity and frontotemporal dementia.. Curr. Alzheimer Res..

[168] Palese F., Bonomi E., Nuzzo T., Benussi A., Mellone M., Zianni E., Cisani F., Casamassa A., Alberici A., Scheggia D., Padovani A., Marcello E., Di Luca M., Pittaluga A., Usiello A., Borroni B., Gardoni F. (2020). Anti-GluA3 antibodies in frontotemporal dementia: Effects on glutamatergic neurotransmission and synaptic failure.. Neurobiol. Aging.

[169] Arshad F., Varghese F., Paplikar A., Gangadhar Y., Ramakrishnan S., Chaudhuri J.R., Mahadevan A., Alladi S. (2021). Role of autoantibodies in neurodegenerative dementia: An emerging association.. Dement. Geriatr. Cogn. Disord..

[170] Maftei M., Thurm F., Schnack C., Tumani H., Otto M., Elbert T., Kolassa I.T., Przybylski M., Manea M., von Arnim C.A.F. (2013). Increased levels of antigen-bound β-amyloid autoantibodies in serum and cerebrospinal fluid of Alzheimer’s disease patients.. PLoS One.

[171] Bartos A., Fialová L., Švarcová J. (2018). Lower serum antibodies against tau protein and heavy neurofilament in alzheimer’s disease.. J. Alzheimers Dis..

[172] Koval L., Lykhmus O., Kalashnyk O., Bachinskaya N., Kravtsova G., Soldatkina M., Zouridakis M., Stergiou C., Tzartos S., Tsetlin V., Komisarenko S., Skok M. (2011). The presence and origin of autoantibodies against α4 and α7 nicotinic acetylcholine receptors in the human blood: Possible relevance to Alzheimer’s pathology.. J. Alzheimers Dis..

[173] Davydova T.V., Mikovskaya O.I., Fomina V.G., Voskresenskaya N.I., Doronina O.A. (2002). Induction of immune complexes and autoantibodies to serotonin and dopamine in patients with Alzheimer’s disease.. Bull. Exp. Biol. Med..

[174] Davydova T.V., Voskresenskaya N.I., Gorbatov V.Y., Fomina V.G., Doronina O.A., Maksunova I.V. (2009). Production of autoantibodies to glutamate during Alzheimer’s dementia.. Bull. Exp. Biol. Med..

[175] Busse S., Brix B., Kunschmann R., Bogerts B., Stoecker W., Busse M. (2014). N-methyl-d-aspartate glutamate receptor (NMDA-R) antibodies in mild cognitive impairment and dementias.. Neurosci. Res..

[176] Gruden M.A., Davidova T.B., Mališauskas M., Sewell R.D.E., Voskresenskaya N.I., Wilhelm K., Elistratova E.I., Sherstnev V.V., Morozova-Roche L.A. (2007). Differential neuroimmune markers to the onset of Alzheimer’s disease neurodegeneration and dementia: Autoantibodies to Aβ(25–35) oligomers, S100b and neurotransmitters.. J. Neuroimmunol..

[177] Mecocci P., Parnetti L., Donato R., Santucci C., Santucci A., Cadini D., Foà E., Cecchetti R., Senin U. (1992). Serum autoantibodies against glial fibrillary acidic protein in brain aging and senile dementias.. Brain Behav. Immun..

[178] McRae A., Dahlström A., Polinsky R., Ling E.A. (1993). Cerebrospinal fluid microglial antibodies: Potential diagnostic markers for immune mechanisms in Alzheimer’s disease.. Behav. Brain Res..

[179] Kingsley B.S., Gaskin F., Fu S.M. (1988). Human antibodies to neurofibrillary tangles and astrocytes in Alzheimer’s disease.. J. Neuroimmunol..

[180] Kankaanpää J., Turunen S.P., Moilanen V., Hörkkö S., Remes A.M. (2009). Cerebrospinal fluid antibodies to oxidized LDL are increased in Alzheimer’s disease.. Neurobiol. Dis..

[181] Vojdani A., Vojdani E. (2018). Amyloid-Beta 1-42 cross-reactive antibody prevalent in human sera may contribute to intraneuronal deposition of A-Beta-P-42.. Int. J. Alzheimers Dis..

[182] Mruthinti S., Schade R., Harrell D., Gulati N., Swamy-Mruthinti S., Lee G., Buccafusco J. (2006). Autoimmunity in Alzheimer’s disease as evidenced by plasma immunoreactivity against RAGE and Abeta42: Complication of diabetes.. Curr. Alzheimer Res..

[183] Giil L.M., Kristoffersen E.K., Vedeler C.A., Aarsland D., Nordrehaug J.E., Winblad B., Cedazo-Minguez A., Lund A., Reksten T.R. (2015). Autoantibodies toward the angiotensin 2 Type 1 receptor: A novel autoantibody in alzheimer’s disease.. J. Alzheimers Dis..

[184] Colasanti T., Barbati C., Rosano G., Malorni W., Ortona E. (2010). Autoantibodies in patients with Alzheimer’s disease: Pathogenetic role and potential use as biomarkers of disease progression.. Autoimmun. Rev..

[185] Ariga T., Jarvis W.D., Yu R.K. (1998). Role of sphingolipid-mediated cell death in neurodegenerative diseases.. J. Lipid Res..

[186] Jianming W., Ling L. (2016). Autoantibodies in Alzheimer’s disease: Potential biomarkers, pathogenic roles, and therapeutic implications.. J. Biomed. Res..

[187] Vacirca D., Delunardo F., Matarrese P., Colasanti T., Margutti P., Siracusano A., Pontecorvo S., Capozzi A., Sorice M., Francia A., Malorni W., Ortona E. (2012). Autoantibodies to the adenosine triphosphate synthase play a pathogenetic role in Alzheimer’s disease.. Neurobiol. Aging.

[188] Berry A., Vacirca D., Capoccia S., Bellisario V., Malorni W., Ortona E., Cirulli F. (2012). Anti-ATP synthase autoantibodies induce neuronal death by apoptosis and impair cognitive performance in C57BL/6J mice.. J. Alzheimers Dis..

[189] Dinkins M.B., Dasgupta S., Wang G., Zhu G., He Q., Kong J.N., Bieberich E. (2015). The 5XFAD mouse model of Alzheimer’s disease exhibits an age-dependent increase in anti-ceramide IgG and exogenous administration of ceramide further increases anti-ceramide titers and amyloid plaque burden.. J. Alzheimers Dis..

[190] Li X., Sundquist J., Sundquist K. (2012). Subsequent risks of Parkinson disease in patients with autoimmune and related disorders: A nationwide epidemiological study from Sweden.. Neurodegener. Dis..

[191] Li X., Sundquist J., Zöller B., Sundquist K. (2018). Dementia and Alzheimer’s disease risks in patients with autoimmune disorders.. Geriatr. Gerontol. Int..

[192] Cho Y.Y., Kim B., Shin D.W., Youn J., Mok J.O., Kim C.H., Kim S.W., Chung J.H., Han K., Kim T.H. (2022). Graves’ disease and the risk of Parkinson’s disease: A Korean population-based study.. Brain Commun..

[193] Bonuccelli U., D’Avino C., Caraccio N., Del Guerra P., Casolaro A., Pavese N., Del Dotto P., Monzani F. (1999). Thyroid function and autoimmunity in Parkinson’s disease: A study of 101 patients.. Parkinsonism Relat. Disord..

[194] Charoenngam N., Rittiphairoj T., Ponvilawan B., Prasongdee K. (2022). Thyroid dysfunction and risk of Parkinson’s disease: A systematic review and meta-analysis.. Front. Endocrinol..

[195] Yeung C.H.C., Au Yeung S.L., Schooling C.M. (2022). Association of autoimmune diseases with Alzheimer’s disease: A mendelian randomization study.. J. Psychiatr. Res..

[196] Ungprasert P., Wijarnpreecha K., Thongprayoon C. (2016). Rheumatoid arthritis and the risk of dementia: A systematic review and meta-analysis.. Neurol. India.

[197] McDowell B., Marr C., Holmes C., Edwards C.J., Cardwell C., McHenry M., Meenagh G., McGuinness B. (2022). Prevalence of cognitive impairment in patients with rheumatoid arthritis: A cross sectional study.. BMC Psychiatry.

[198] Tansey M.G., Wallings R.L., Houser M.C., Herrick M.K., Keating C.E., Joers V. (2022). Inflammation and immune dysfunction in Parkinson disease.. Nat. Rev. Immunol..

[199] Li D., Hong X., Chen T. (2022). Association between rheumatoid arthritis and risk of Parkinson’s disease: A meta-analysis and systematic review.. Front. Neurol..

[200] Li M., Wan J., Xu Z., Tang B. (2023). The association between Parkinson’s disease and autoimmune diseases: A systematic review and meta-analysis.. Front. Immunol..

[201] Policicchio S., Ahmad A.N., Powell J.F., Proitsi P. (2017). Rheumatoid arthritis and risk for Alzheimer’s disease: A systematic review and meta-analysis and a Mendelian Randomization study.. Sci. Rep..

[202] Cooper J., Pastorello Y., Slevin M. (2023). A meta-analysis investigating the relationship between inflammation in autoimmune disease, elevated CRP, and the risk of dementia.. Front. Immunol..

[203] Karabay E.A., Çerman A.A.; (2018). Altunay, İ.K. Evaluation of comorbidities in patients with autoimmune bullous diseases: A retrospective study.. Sisli Etfal Hastan Tip Bul..

[204] Yeh F.C., Chen H.C., Chou Y.C., Lin C.L., Kao C.H., Lo H.Y., Liu F.C., Yang T.Y. (2020). Positive association of Parkinson’s disease with ankylosing spondylitis: A nationwide population-based study.. J. Transl. Med..

[205] Rønnow Sand J., Troelsen F.S., Horváth-Puhó E., Henderson V.W., Sørensen H.T., Erichsen R. (2022). Risk of dementia in patients with inflammatory bowel disease: A Danish population-based study.. Aliment. Pharmacol. Ther..

[206] Zhang B., Wang H.E., Bai Y.M., Tsai S.J., Su T.P., Chen T.J., Wang Y.P., Chen M.H. (2021). Inflammatory bowel disease is associated with higher dementia risk: A nationwide longitudinal study.. Gut.

[207] Szandruk-Bender M., Wiatrak B.; (2022). Szeląg, A. The risk of developing Alzheimer’s disease and Parkinson’s disease in patients with inflammatory bowel disease: A meta-analysis.. J. Clin. Med..

[208] Aggarwal M., Alkhayyat M., Abou Saleh M., Sarmini M.T., Singh A., Garg R., Garg P., Mansoor E., Padival R., Cohen B.L. (2023). Alzheimer disease occurs more frequently in patients with inflammatory bowel disease.. J. Clin. Gastroenterol..

[209] Cui G., Li S., Ye H., Yang Y., Huang Q., Chu Y., Shi Z., Zhang X. (2022). Are neurodegenerative diseases associated with an increased risk of inflammatory bowel disease? A two-sample Mendelian randomization study.. Front. Immunol..

[210] Li H., Wen Z. (2022). Effects of ulcerative colitis and Crohn’s disease on neurodegenerative diseases: A Mendelian randomization study.. Front. Genet..

[211] Freuer D., Meisinger C. (2022). Association between inflammatory bowel disease and Parkinson’s disease: A Mendelian randomization study.. NPJ Parkinsons Dis..

[212] Huang J., Su B., Karhunen V., Gill D., Zuber V., Ahola-Olli A., Palaniswamy S., Auvinen J., Herzig K.H., Keinänen-Kiukaanniemi S., Salmi M., Jalkanen S., Lehtimäki T., Salomaa V., Raitakari O.T., Matthews P.M., Elliott P., Tsilidis K.K., Jarvelin M., Tzoulaki I., Dehghan A. (2023). Inflammatory diseases, inflammatory biomarkers, and Alzheimer disease.. Neurology.

[213] Liu F.C., Huang W.Y., Lin T.Y., Shen C.H., Chou Y.C., Lin C.L., Lin K.T., Kao C.H. (2015). Inverse association of Parkinson disease with systemic lupus erythematosus.. Medicine.

[214] Wang Y.C., Lin M.S., Huang A.P.H., Wu C.C., Kung W.M. (2022). Association between systemic rheumatic diseases and dementia risk: A meta-analysis.. Front. Immunol..

[215] Jin T., Huang W., Cao F., Yu X., Guo S., Ying Z., Xu C. (2022). Causal association between systemic lupus erythematosus and the risk of dementia: A Mendelian randomization study.. Front. Immunol..

[216] Chen H., Zhang S.M., Hernán M.A., Schwarzschild M.A., Willett W.C., Colditz G.A., Speizer F.E., Ascherio A. (2003). Nonsteroidal anti-inflammatory drugs and the risk of Parkinson disease.. Arch. Neurol..

[217] Chen H., Jacobs E., Schwarzschild M.A., McCullough M.L., Calle E.E., Thun M.J., Ascherio A. (2005). Nonsteroidal antiinflammatory drug use and the risk for Parkinson’s disease.. Ann. Neurol..

[218] Gagne J.J., Power M.C. (2010). Anti-inflammatory drugs and risk of Parkinson disease: A meta-analysis.. Neurology.

[219] Gao X., Chen H., Schwarzschild M.A., Ascherio A. (2011). Use of ibuprofen and risk of Parkinson disease.. Neurology.

[220] Powers K.M., Kay D.M., Factor S.A., Zabetian C.P., Higgins D.S., Samii A., Nutt J.G., Griffith A., Leis B., Roberts J.W., Martinez E.D., Montimurro J.S., Checkoway H., Payami H. (2008). Combined effects of smoking, coffee, and NSAIDs on Parkinson’s disease risk.. Mov. Disord..

[221] San Luciano M., Tanner C.M., Meng C., Marras C., Goldman S.M., Lang A.E., Tolosa E., Schüle B., Langston J.W., Brice A., Corvol J.C., Goldwurm S., Klein C., Brockman S., Berg D., Brockmann K., Ferreira J.J., Tazir M., Mellick G.D., Sue C.M., Hasegawa K., Tan E.K., Bressman S., Saunders-Pullman R., Saunders-Pullman R., Raymond D., Deik A., Barrett M.J., Cabassa J., Groves M., Hunt A.L., Lubarr N., Miravite J., Palmese C., Sachdev R., Sarva H., Severt L., Shanker V., Swan M.C., Soto-Valencia J., Johannes B., Ortega R., Ozelius L., Bressman S., Alcalay R.N., Tang M-X., Santana H.M., Roos E., Orbe-Reilly M., Fahn S., Cote L., Waters C., Mazzoni P., Ford B., Louis E., Levy O., Rosado L., Ruiz D., Dorovski T., Clark L., Marder K.S., Corvol J-C., Cormier F., Bonnet A-M., Welter M-L., Mesnage V., Vidailhet M., Roze E., Lacomblez L., Grabli D., Mart i Masso J.F., Martinez J.R., Mondragon R.E., Alustiza A.E., Pagola A.G., Pont-Sunyer C., Rolan D.V., Fernandez-Santiago R., Quintana M., Fernandez M., Maragall L., Hentati F., Farrer M., Duda J., Read M., Middleton L., Gibson R., Trinh J., Sassi S.B., Zouari M.; (2020). Rimamouri,; Farhat, E.; Nabli, F.; Aasly, J.; Warø, B.J.; Andersen, S.; Bertoni, J.; Carter, J.; Elmer, L.; Jimenez, N.G.; Martin, W.; Pahwa, R.; Lyons, K.; Reich, S.; Rodnitzky, R.; Ramos, C.S.; Wojcieszek, J.; Mirelman, A.; Gurevich, T.; Shira, A.B.; Weisz, M.G.; Yasinovsky, K.; Zalis, M.; Thaler, A.; Orr-Urtreger, A.; Giladi, N.; Mountain, J.; Mestre, T.; Visanji, N.; Ghate, T.; Singerman, J.; Al Dakheel, A.; Connolly, B.S.; Gasser, T.; Brockmann, K.; Conley, E.D.; Mullins, M.E.; Northover, C.; Facheris, M.; Fiske, B.; Urkowiz, A. Nonsteroidal anti-inflammatory use and LRRK2 Parkinson’s disease penetrance.. Mov. Disord..

[222] Ren L., Yi J., Yang J., Li P., Cheng X., Mao P. (2018). Nonsteroidal anti-inflammatory drugs use and risk of Parkinson disease.. Medicine.

[223] Brakedal B., Tzoulis C., Tysnes O.B., Haugarvoll K. (2021). NSAID use is not associated with Parkinson’s disease incidence: A Norwegian Prescription Database study.. PLoS One.

[224] Chou R.C., Kane M., Ghimire S., Gautam S., Gui J. (2016). Treatment for rheumatoid arthritis and risk of Alzheimer’s disease: A nested case/control analysis.. CNS Drugs.

[225] Zhou M., Xu R., Kaelber D.C., Gurney M.E. (2020). Tumor Necrosis Factor (TNF) blocking agents are associated with lower risk for Alzheimer’s disease in patients with rheumatoid arthritis and psoriasis.. PLoS One.

[226] Zheng C., Fillmore N.R., Ramos-Cejudo J., Brophy M., Osorio R., Gurney M.E., Qiu W.Q., Au R., Perry G., Dubreuil M., Chen S.G., Qi X., Davis P.B., Do N., Xu R. (2022). Potential long-term effect of tumor necrosis factor inhibitors on dementia risk: A propensity score matched retrospective cohort study in US veterans.. Alzheimers Dement..

[227] Newby D., Prieto-Alhambra D., Duarte-Salles T., Ansell D., Pedersen L., van der Lei J., Mosseveld M., Rijnbeek P., James G., Alexander M., Egger P., Podhorna J., Stewart R., Perera G., Avillach P., Grosdidier S., Lovestone S., Nevado-Holgado A.J. (2020). Methotrexate and relative risk of dementia amongst patients with rheumatoid arthritis: A multi-national multi-database case-control study.. Alzheimers Res. Ther..

[228] Watad A., McGonagle D., Anis S., Carmeli R., Cohen A.D., Tsur A.M., Ben-Shabat N., Luigi Bragazzi N., Lidar M., Amital H. (2022). TNF inhibitors have a protective role in the risk of dementia in patients with ankylosing spondylitis: Results from a nationwide study.. Pharmacol. Res..

[229] Peter I., Dubinsky M., Bressman S., Park A., Lu C., Chen N., Wang A. (2018). Anti–tumor necrosis factor therapy and incidence of Parkinson disease among patients with inflammatory bowel disease.. JAMA Neurol..

[230] Kern D.M., Lovestone S., Cepeda M.S. (2021). Treatment with TNF-α inhibitors versus methotrexate and the association with dementia and Alzheimer’s disease.. Alzheimers Dement..

[231] Desai R.J., Varma V.R., Gerhard T., Segal J., Mahesri M., Chin K., Horton D.B., Kim S.C., Schneeweiss S., Thambisetty M. (2022). Comparative risk of Alzheimer disease and related dementia among Medicare beneficiaries with Rheumatoid Arthritis treated with targeted disease/modifying antirheumatic agents.. JAMA Netw. Open.

[232] Fardet L., Nazareth I., Petersen I. (2019). Chronic hydroxychloroquine/chloroquine exposure for connective tissue diseases and risk of Alzheimer’s disease: A population-based cohort study.. Ann. Rheum. Dis..

[233] Lai S.W., Kuo Y.H., Liao K.F. (2021). Chronic hydroxychloroquine exposure and the risk of Alzheimer’s disease.. Ann. Rheum. Dis..

[234] Varma V.R., Desai R.J., Navakkode S., Wong L.W., Anerillas C., Loeffler T., Schilcher I., Mahesri M., Chin K., Horton D.B., Kim S.C., Gerhard T., Segal J.B., Schneeweiss S., Gorospe M., Sajikumar S., Thambisetty M. (2023). Hydroxychloroquine lowers Alzheimer’s disease and related dementias risk and rescues molecular phenotypes related to Alzheimer’s disease.. Mol. Psychiatry.

[235] Mathieu S., Couderc M., Pereira B., Dubost J.J., Malochet-Guinamand S., Tournadre A., Soubrier M., Moisset X. (2020). Prevalence of migraine and neuropathic pain in rheumatic diseases.. J. Clin. Med..

[236] Wu L., Xu Q., Zhou M., Chen Y., Jiang C., Jiang Y., Lin Y., He Q., Zhao L., Dong Y., Liu J., Chen W. (2022). Plasma miR-153 and miR-223 levels as potential biomarkers in Parkinson’s disease.. Front. Neurosci..

[237] Li D., Yang H., Ma J., Luo S., Chen S., Gu Q. (2018). MicroRNA-30e regulates neuroinflammation in MPTP model of Parkinson’s disease by targeting Nlrp3.. Hum. Cell.

[238] Taglialatela G., Rastellini C., Cicalese L. (2015). Reduced incidence of dementia in solid organ transplant patients treated with calcineurin inhibitors.. J. Alzheimers Dis..

[239] Bukhbinder A.S., Ling Y., Hasan O., Jiang X., Kim Y., Phelps K.N., Schmandt R.E., Amran A., Coburn R., Ramesh S., Xiao Q., Schulz P.E. (2022). Risk of Alzheimer’s disease following influenza vaccination: A claims-based cohort study using propensity score matching.. J. Alzheimers Dis..

[240] Klinger D., Hill B.L., Barda N., Halperin E., Gofrit O.N., Greenblatt C.L., Rappoport N., Linial M., Bercovier H. (2021). Bladder cancer immunotherapy by BCG is associated with a significantly reduced risk of Alzheimer’s disease and Parkinson’s disease.. Vaccines,.

[241] Al-kuraishy H.M., Al-Gareeb A.I., Saad H.M., Batiha G.E.S. (2023). Long-term use of metformin and Alzheimer’s disease: Beneficial or detrimental effects.. Inflammopharmacology.

[242] McGeer P.L., Rogers J., McGeer E.G. (2006). Inflammation, anti-inflammatory agents and Alzheimer disease: The last 12 years.. J. Alzheimers Dis..

[243] Launer L.J. (2003). Nonsteroidal anti-inflammatory drug use and the risk for Alzheimer’s disease: dissecting the epidemiological evidence.. Drugs.

[244] Daniels M.J.D., Rivers-Auty J., Schilling T., Spencer N.G., Watremez W., Fasolino V., Booth S.J., White C.S., Baldwin A.G., Freeman S., Wong R., Latta C., Yu S., Jackson J., Fischer N., Koziel V., Pillot T., Bagnall J., Allan S.M., Paszek P., Galea J., Harte M.K., Eder C., Lawrence C.B., Brough D. (2016). Fenamate NSAIDs inhibit the NLRP3 inflammasome and protect against Alzheimer’s disease in rodent models.. Nat. Commun..

[245] Annadurai N., De Sanctis J.B., Hajdúch M., Das V. (2021). Tau secretion and propagation: Perspectives for potential preventive interventions in Alzheimer’s disease and other tauopathies.. Exp. Neurol..

[246] Annadurai N., Malina L., Malohlava J., Hajdúch M., Das V. (2022). Tau R2 and R3 are essential regions for tau aggregation, seeding and propagation.. Biochimie.

[247] Annadurai N., Malina L., Salmona M., Diomede L., Bastone A., Cagnotto A., Romeo M., Šrejber M., Berka K., Otyepka M., Hajdúch M., Das V. (2022). Antitumour drugs targeting tau R3 VQIVYK and Cys322 prevent seeding of endogenous tau aggregates by exogenous seeds.. FEBS J..

[248] Annadurai N., Hrubý J.; (2023). Kubíčková, A.; Malina, L.; Hajdúch, M.; Das, V. Time- and dose-dependent seeding tendency of exogenous tau R2 and R3 aggregates in cells.. Biochem. Biophys. Res. Commun..

[249] Ferretti M.T., Allard S., Partridge V., Ducatenzeiler A., Cuello A.C. (2012). Minocycline corrects early, pre-plaque neuroinflammation and inhibits BACE-1 in a transgenic model of Alzheimer’s disease-like amyloid pathology.. J. Neuroinflammation.

[250] Parashos S.A., Luo S., Biglan K.M., Bodis-Wollner I., He B., Liang G.S., Ross G.W., Tilley B.C., Shulman L.M. (2014). Measuring disease progression in early Parkinson disease.. JAMA Neurol..

[251] Nassar N.N., Al-Shorbagy M.Y., Arab H.H., Abdallah D.M. (2015). Saxagliptin: A novel antiparkinsonian approach.. Neuropharmacology.

[252] Chen S., Zhou M., Sun J., Guo A., Fernando R.L., Chen Y., Peng P., Zhao G., Deng Y. (2019). DPP-4 inhibitor improves learning and memory deficits and AD-like neurodegeneration by modulating the GLP-1 signaling.. Neuropharmacology.

[253] Yu H., Sun T., He X., Wang Z., Zhao K., An J., Wen L., Li J.Y., Li W., Feng J. (2022). Association between Parkinson’s disease and diabetes mellitus: From epidemiology, pathophysiology and prevention to treatment.. Aging Dis..

[254] Landreth G., Jiang Q., Mandrekar S., Heneka M. (2008). PPARγ agonists as therapeutics for the treatment of Alzheimer’s disease.. Neurotherapeutics.

[255] Watson G.S., Cholerton B.A., Reger M.A., Baker L.D., Plymate S.R., Asthana S., Fishel M.A., Kulstad J.J., Green P.S., Cook D.G., Kahn S.E., Keeling M.L., Craft S. (2005). Preserved cognition in patients with early Alzheimer disease and amnestic mild cognitive impairment during treatment with rosiglitazone: A preliminary study.. Am. J. Geriatr. Psychiatry.

[256] Risner M.E., Saunders A.M., Altman J F B., Ormandy G.C., Craft S., Foley I.M., Zvartau-Hind M.E., Hosford D.A., Roses A.D. (2006). Efficacy of rosiglitazone in a genetically defined population with mild-to-moderate Alzheimer’s disease.. Pharmacogenomics J..

[257] Alhowail A., Alsikhan R., Alsaud M., Aldubayan M., Rabbani S.I. (2022). Protective effects of pioglitazone on cognitive impairment and the underlying mechanisms: A review of literature.. Drug Des. Devel. Ther..

[258] Zhou Y., Chen Y., Xu C., Zhang H., Lin C. (2020). TLR4 targeting as a promising therapeutic strategy for Alzheimer disease treatment.. Front. Neurosci..

[259] Cui W., Sun C., Ma Y., Wang S., Wang X., Zhang Y. (2020). Inhibition of TLR4 Induces M2 microglial polarization and provides neuroprotection via the NLRP3 inflammasome in Alzheimer’s disease.. Front. Neurosci..

[260] Jin X., Liu M.Y., Zhang D.F., Zhong X., Du K., Qian P., Yao W.F., Gao H., Wei M.J. (2019). Baicalin mitigates cognitive impairment and protects neurons from microglia-mediated neuroinflammation via suppressing NLRP 3 inflammasomes and TLR 4/NFκB signaling pathway.. CNS Neurosci. Ther..

[261] Shi S., Liang D., Chen Y., Xie Y., Wang Y., Wang L., Wang Z., Qiao Z. (2016). Gx-50 reduces β-amyloid-induced TNF-α IL-1β NO, and PGE2 expression and inhibits NF-κB signaling in a mouse model of Alzheimer’s disease.. Eur. J. Immunol..

[262] Kim C., Spencer B., Rockenstein E., Yamakado H., Mante M., Adame A., Fields J.A., Masliah D., Iba M., Lee H.J., Rissman R.A., Lee S.J., Masliah E. (2018). Immunotherapy targeting toll-like receptor 2 alleviates neurodegeneration in models of synucleinopathy by modulating α-synuclein transmission and neuroinflammation.. Mol. Neurodegener..

[263] Lee H., Jeon S.G., Kim J., Kang R.J., Kim S.M., Han K.M., Park H., Kim K., Sung Y.M., Nam H.Y., Koh Y.H., Song M., Suk K., Hoe H.S. (2021). Ibrutinib modulates Aβ/tau pathology, neuroinflammation, and cognitive function in mouse models of Alzheimer’s disease.. Aging Cell.

[264] He P., Cheng X., Staufenbiel M., Li R., Shen Y. (2013). Long-term treatment of thalidomide ameliorates amyloid-like pathology through inhibition of β-secretase in a mouse model of Alzheimer’s disease.. PLoS One.

[265] Decourt B., Drumm-Gurnee D., Wilson J., Jacobson S., Belden C., Sirrel S., Ahmadi M., Shill H., Powell J., Walker A., Gonzales A., Macias M., Sabbagh M.N. (2017). Poor safety and tolerability hamper reaching a potentially therapeutic dose in the use of thalidomide for Alzheimer’s disease: Results from a double-blind, placebo-controlled trial.. Curr. Alzheimer Res..

[266] Decourt B., Wilson J., Ritter A., Dardis C., DiFilippo F., Zhuang X., Cordes D., Lee G., Fulkerson N., St Rose T., Hartley K., Sabbagh M. (2020). MCLENA-1: A phase ii clinical trial for the assessment of safety, tolerability, and efficacy of lenalidomide in patients with mild cognitive impairment due to Alzheimer’s disease.. Open Access J. Clin. Trials.

[267] Palmas M.F., Ena A., Burgaletto C., Casu M.A., Cantarella G., Carboni E., Etzi M., De Simone A., Fusco G., Cardia M.C., Lai F., Picci L., Tweedie D., Scerba M.T., Coroneo V., Bernardini R., Greig N.H., Pisanu A., Carta A.R. (2022). Repurposing pomalidomide as a neuroprotective drug: Efficacy in an alpha-synuclein-based model of parkinson’s disease.. Neurotherapeutics.

[268] Singh S., Ganguly U., Pal S., Chandan G., Thakur R., Saini R.V., Chakrabarti S.S., Agrawal B.K., Chakrabarti S. (2022). Protective effects of cyclosporine A on neurodegeneration and motor impairment in rotenone-induced experimental models of Parkinson’s disease.. Eur. J. Pharmacol..

[269] Van der Perren A., Macchi F., Toelen J., Carlon M.S., Maris M., de Loor H., Kuypers D.R.J., Gijsbers R., Van den Haute C., Debyser Z., Baekelandt V. (2015). FK506 reduces neuroinflammation and dopaminergic neurodegeneration in an α-synuclein-based rat model for Parkinson’s disease.. Neurobiol. Aging.

[270] Köylü A., Altunkaynak B.Z.; (2021). Delibaş, B. Effects of tacrolimus on c-fos in hippocampus and memory performances in streptozotocin model of Alzheimer’s disease of rats.. Turk. J. Med. Sci..

[271] Kumar A., Singh N. (2018). Calcineurin inhibition and protein kinase a activation limits cognitive dysfunction and histopathological damage in a model of dementia of the Alzheimer’s type.. Curr. Neurovasc. Res..

[272] Lai W.D., Wang S., You W.T., Chen S.J., Wen J.J., Yuan C.R., Zheng M.J., Jin Y., Yu J., Wen C.P. (2022). Sinomenine regulates immune cell subsets: Potential neuro-immune intervene for precise treatment of chronic pain.. Front. Cell Dev. Biol..

[273] Alam J., Blackburn K., Patrick D. (2017). Neflamapimod: Clinical phase 2b-ready oral small molecule inhibitor of p38α to reverse synaptic dysfunction in early Alzheimer’s disease.. J. Prev. Alzheimers Dis..

[274] Prins N.D., Harrison J.E., Chu H.M., Blackburn K., Alam J.J., Scheltens P. (2021). A phase 2 double-blind placebo-controlled 24-week treatment clinical study of the p38 alpha kinase inhibitor neflamapimod in mild Alzheimer’s disease.. Alzheimers Res. Ther..

[275] Rothhammer V., Kenison J.E., Li Z., Tjon E., Takenaka M.C., Chao C.C., Alves de Lima K., Borucki D.M., Kaye J., Quintana F.J. (2021). Aryl hydrocarbon receptor activation in astrocytes by laquinimod ameliorates autoimmune inflammation in the CNS.. Neurol. Neuroimmunol. Neuroinflamm..

[276] Srivastava S., Rajopadhye R., Dey M., Singh R.K. (2021). Inhibition of MK2 kinase as a potential therapeutic target to control neuroinflammation in Alzheimer’s disease.. Expert Opin. Ther. Targets.

[277] Roy S.M., Minasov G., Arancio O., Chico L.W., Van Eldik L.J., Anderson W.F., Pelletier J.C., Watterson D.M. (2019). A selective and brain penetrant p38αMAPK inhibitor candidate for neurologic and neuropsychiatric disorders that attenuates neuroinflammation and cognitive dysfunction.. J. Med. Chem..

[278] Martínez G., Mijares M.R., De Sanctis J.B. (2019). Effects of flavonoids and its derivatives on immune cell responses.. Recent Pat. Inflamm. Allergy Drug Discov..

[279] Ping Z., Xiaomu W., Xufang X., Liang S. (2019). Vinpocetine regulates levels of circulating TLRs in Parkinson’s disease patients.. Neurol. Sci..

[280] Cui B., Guo X., You Y., Fu R. (2019). Farrerol attenuates MPP+induced inflammatory response by TLR4 signaling in a microglia cell line.. Phytother. Res..

[281] Yang Y.L., Cheng X., Li W.H., Liu M., Wang Y.H., Du G.H. (2019). Kaempferol attenuates LPS-induced striatum injury in mice involving anti-neuroinflammation, maintaining BBB integrity, and down-regulating the HMGB1/TLR4 pathway.. Int. J. Mol. Sci..

[282] Yang L., Zhou R., Tong Y., Chen P., Shen Y., Miao S., Liu X. (2020). Neuroprotection by dihydrotestosterone in LPS-induced neuroinflammation.. Neurobiol. Dis..

[283] Haddadi R., Nayebi A.M., Eyvari B.S. (2018). RETRACTED: Silymarin prevents apoptosis through inhibiting the Bax/caspase-3 expression and suppresses toll like receptor-4 pathway in the SNc of 6-OHDA intoxicated rats.. Biomed. Pharmacother..

[284] Su Q., Ng W.L., Goh S.Y., Gulam M.Y., Wang L.F., Tan E.K., Ahn M., Chao Y.X. (2022). Targeting the inflammasome in Parkinson’s disease.. Front. Aging Neurosci..

[285] Yang Y., Guo L., Wang J., Li W., Zhou X., Zhang C., Han C. (2022). Arglabin regulates microglia polarization to relieve neuroinflammation in Alzheimer’s disease.. J. Biochem. Mol. Toxicol..

[286] Tong B.C.K., Huang A.S., Wu A.J., Iyaswamy A., Ho O.K.Y., Kong A.H.Y., Sreenivasmurthy S.G., Zhu Z., Su C., Liu J., Song J., Li M., Cheung K.H. (2022). Tetrandrine ameliorates cognitive deficits and mitigates tau aggregation in cell and animal models of tauopathies.. J. Biomed. Sci..

[287] Velagapudi R., Aderogba M., Olajide O.A. (2014). Tiliroside, a dietary glycosidic flavonoid, inhibits TRAF-6/NF-κB/p38-mediated neuroinflammation in activated BV2 microglia.. Biochim. Biophys. Acta, Gen. Subj..

[288] Wu Q., Naeem A., Zou J., Yu C., Wang Y., Chen J., Ping Y. (2022). Isolation of phenolic compounds from raspberry based on molecular imprinting techniques and investigation of their anti-alzheimer’s disease properties.. Molecules.

[289] Rezai-Zadeh K., Ehrhart J., Bai Y., Sanberg P.R., Bickford P., Tan J., Shytle R.D. (2008). Apigenin and luteolin modulate microglial activation via inhibition of STAT1-induced CD40 expression.. J. Neuroinflammation.

[290] Liu R., Zhang T., Yang H., Lan X., Ying J., Du G. (2011). The flavonoid apigenin protects brain neurovascular coupling against amyloid-β₂₅₋₃₅-induced toxicity in mice.. J. Alzheimers Dis..

[291] Kang C.H., Choi Y.H., Moon S.K., Kim W.J., Kim G.Y. (2013). Quercetin inhibits lipopolysaccharide-induced nitric oxide production in BV2 microglial cells by suppressing the NF-κB pathway and activating the Nrf2-dependent HO-1 pathway.. Int. Immunopharmacol..

[292] Wightman E.L., Haskell C.F., Forster J.S., Veasey R.C., Kennedy D.O. (2012). Epigallocatechin gallate, cerebral blood flow parameters, cognitive performance and mood in healthy humans: a double-blind, placebo-controlled, crossover investigation.. Hum. Psychopharmacol..

[293] Olajide O.A., Sarker S.D. (2020). Alzheimer’s disease: Natural products as inhibitors of neuroinflammation.. Inflammopharmacology.

[294] Moussa C., Hebron M., Huang X., Ahn J., Rissman R.A., Aisen P.S., Turner R.S. (2017). Resveratrol regulates neuro-inflammation and induces adaptive immunity in Alzheimer’s disease.. J. Neuroinflammation.

[295] Porro C., Cianciulli A., Trotta T., Lofrumento D.D., Panaro M.A. (2019). Curcumin regulates anti-inflammatory responses by JAK/STAT/SOCS signaling pathway in bv-2 microglial cells.. Biology,.

[296] Sorrenti V., Contarini G., Sut S., Dall’Acqua S., Confortin F., Pagetta A., Giusti P., Zusso M. (2018). Curcumin prevents acute neuroinflammation and long-term memory impairment induced by systemic lipopolysaccharide in mice.. Front. Pharmacol..

[297] Sundaram J.R., Poore C.P., Sulaimee N.H.B., Pareek T., Cheong W.F., Wenk M.R., Pant H.C., Frautschy S.A., Low C.M., Kesavapany S. (2017). Curcumin ameliorates neuroinflammation, neurodegeneration, and memory deficits in p25 transgenic mouse model that bears hallmarks of alzheimer’s disease.. J. Alzheimers Dis..

[298] Ringman J.M., Frautschy S.A., Teng E., Begum A.N., Bardens J., Beigi M., Gylys K.H., Badmaev V., Heath D.D., Apostolova L.G., Porter V., Vanek Z., Marshall G.A., Hellemann G., Sugar C., Masterman D.L., Montine T.J., Cummings J.L., Cole G.M. (2012). Oral curcumin for Alzheimer’s disease: Tolerability and efficacy in a 24-week randomized, double blind, placebo-controlled study.. Alzheimers Res. Ther..

[299] Cox K.H.M., Pipingas A., Scholey A.B. (2015). Investigation of the effects of solid lipid curcumin on cognition and mood in a healthy older population.. J. Psychopharmacol..

[300] Small G.W., Siddarth P., Li Z., Miller K.J., Ercoli L., Emerson N.D., Martinez J., Wong K.P., Liu J., Merrill D.A., Chen S.T., Henning S.M., Satyamurthy N., Huang S.C., Heber D., Barrio J.R. (2018). Memory and brain amyloid and tau effects of a bioavailable form of curcumin in non-demented adults: A double-blind, placebo-controlled 18-month trial.. Am. J. Geriatr. Psychiatry.

[301] Khare P., Datusalia A.K., Sharma S.S. (2017). Parthenolide, an NF-κB Inhibitor ameliorates diabetes-induced behavioural deficit, neurotransmitter imbalance and neuroinflammation in type 2 diabetes rat model.. Neuromol. Med..

[302] Qiang W., Cai W., Yang Q., Yang L., Dai Y., Zhao Z., Yin J., Li Y., Li Q., Wang Y., Weng X., Zhang D., Chen Y., Zhu X., Artemisinin B., Artemisinin B. (2018). Improves learning and memory impairment in AD dementia mice by suppressing neuroinflammation.. Neuroscience.

[303] Zhou J.M., Gu S.S., Mei W.H., Zhou J., Wang Z.Z., Xiao W. (2016). Ginkgolides and bilobalide protect BV2 microglia cells against OGD/reoxygenation injury by inhibiting TLR2/4 signaling pathways.. Cell Stress Chaperones.

[304] de Oliveira M.R. (2016). The dietary components carnosic acid and carnosol as neuroprotective agents: A Mechanistic View.. Mol. Neurobiol..

[305] Velagapudi R., Kumar A., Bhatia H.S., El-Bakoush A., Lepiarz I., Fiebich B.L., Olajide O.A. (2017). Inhibition of neuroinflammation by thymoquinone requires activation of Nrf2/ARE signalling.. Int. Immunopharmacol..

[306] Yang W., Qiu X., Wu Q., Chang F., Zhou T., Zhou M., Pei J. (2023). Active constituents of saffron (Crocus sativus L.) and their prospects in treating neurodegenerative diseases. (Review).. Exp. Ther. Med..

[307] Fu M., Liang X., Zhang X., Yang M., Ye Q., Qi Y., Liu H., Zhang X. (2023). Astaxanthin delays brain aging in senescence-accelerated mouse prone 10: inducing autophagy as a potential mechanism.. Nutr. Neurosci..

[308] Lin C.H., Chou C.C., Lee Y.H., Hung C.C. (2022). Curcumin facilitates aryl hydrocarbon receptor activation to ameliorate inflammatory astrogliosis.. Molecules.

[309] Hong S., Beja-Glasser V.F., Nfonoyim B.M., Frouin A., Li S., Ramakrishnan S., Merry K.M., Shi Q., Rosenthal A., Barres B.A., Lemere C.A., Selkoe D.J., Stevens B. (2016). Complement and microglia mediate early synapse loss in Alzheimer mouse models.. Science.

[310] Pittock S.J., Berthele A., Fujihara K., Kim H.J., Levy M., Palace J., Nakashima I., Terzi M., Totolyan N., Viswanathan S., Wang K.C., Pace A., Fujita K.P., Armstrong R., Wingerchuk D.M. (2019). Eculizumab in aquaporin-4-positive neuromyelitis optica spectrum disorder.. N. Engl. J. Med..

[311] Lamers C., Mastellos D.C., Ricklin D., Lambris J.D. (2022). Compstatins: The dawn of clinical C3-targeted complement inhibition.. Trends Pharmacol. Sci..

[312] Lansita J.A., Mease K.M., Qiu H., Yednock T., Sankaranarayanan S., Kramer S. (2017). Nonclinical development of ANX005: A humanized anti-C1q antibody for treatment of autoimmune and neurodegenerative diseases.. Int. J. Toxicol..

[313] Qi Y., Klyubin I., Cuello A.C., Rowan M.J. (2018). NLRP3-dependent synaptic plasticity deficit in an Alzheimer’s disease amyloidosis model in vivo.. Neurobiol. Dis..

[314] Ben-Menachem-Zidon O., Ben-Menahem Y., Ben-Hur T., Yirmiya R. (2014). Intra-hippocampal transplantation of neural precursor cells with transgenic over-expression of IL-1 receptor antagonist rescues memory and neurogenesis impairments in an Alzheimer’s disease model.. Neuropsychopharmacology.

[315] Cavanagh C., Tse Y.C., Nguyen H.B., Krantic S., Breitner J.C.S., Quirion R., Wong T.P. (2016). Inhibiting tumor necrosis factor-α before amyloidosis prevents synaptic deficits in an Alzheimer’s disease model.. Neurobiol. Aging.

[316] Cavanagh C., Wong T.P. (2018). Preventing synaptic deficits in Alzheimer’s disease by inhibiting tumor necrosis factor alpha signaling.. IBRO Rep..

[317] Li Y., Fan H., Ni M., Zhang W., Fang F., Sun J., Lyu P., Ma P. (2022). Etanercept reduces neuron injury and neuroinflammation via inactivating c-Jun N-terminal kinase and nuclear factor-κB Pathways in Alzheimer’s disease: An in vitro and in vivo investigation.. Neuroscience.

[318] Tobinick E., Gross H., Weinberger A., Cohen H. (2006). TNF-alpha modulation for treatment of Alzheimer’s disease: A 6-month pilot study.. MedGenMed.

[319] Tobinick E.L., Gross H. (2008). Rapid improvement in verbal fluency and aphasia following perispinal etanercept in Alzheimer’s disease.. BMC Neurol..

[320] Butchart J., Brook L., Hopkins V., Teeling J., Püntener U., Culliford D., Sharples R., Sharif S., McFarlane B., Raybould R., Thomas R., Passmore P., Perry V.H., Holmes C. (2015). Etanercept in Alzheimer disease: A randomized, placebo-controlled, double-blind, phase 2 trial.. Neurology.

[321] Tufan A.N., Holmes C., Tufan F. (2015). Etanercept in Alzheimer disease: A randomized, placebo-controlled, double-blind, phase 2 trialAuthor Response.. Neurology.

[322] Torres-Acosta N., O’Keefe J.H., O’Keefe E.L., Isaacson R., Small G. (2020). Therapeutic potential of TNF-α inhibition for Alzheimer’s disease prevention.. J. Alzheimers Dis..

[323] vom Berg J., Prokop S., Miller K.R., Obst J., Kälin R.E., Lopategui-Cabezas I., Wegner A., Mair F., Schipke C.G., Peters O., Winter Y., Becher B., Heppner F.L. (2012). Inhibition of IL-12/IL-23 signaling reduces Alzheimer’s disease-like pathology and cognitive decline.. Nat. Med..

[324] Pedrini S., Gupta V.B., Hone E., Doecke J., O’Bryant S., James I., Bush A.I., Rowe C.C., Villemagne V.L., Ames D., Masters C.L., Martins R.N., Savage G., Wilson B., Bourgeat P., Fripp J., Gibson S., Leroux H., McBride S., Salvado O., Fenech M., Francois M., Barnes M., Baker J., Barnham K., Bellingham S., Bomke J., Pejoska S.B., Buckley R., Cheng L., Collins S., Cooke I., Cyarto E., Darby D., Dore V., El-Sheikh D., Faux N., Fowler C., Harrington K., Hill A., Horne M., Jones G., Kamer A., Killeen N., Korrel H., Lamb F., Lautenschlager N., Lennon K., Li Q-X., Lim Y.Y., Louey A., Macaulay L., Mackintosh L., Maruff P., Mcilroy A., Nigro J., Perez K., Pertile K., Restrepo C., Cardoso B.R., Rembach A., Roberts B., Robertson J., Rumble R., Ryan T., Sach J., Silbert B., Thai C., Trounson B., Volitakis I., Vovos M., Ward L., Watt A., Williams R., Woodward M., Yates P., Ugarte F.Y., Zhang P., Bird S., Brown B., Burnham S., Chatterjee P., Cox K., Fernandez S., Fernando B., Gardener S., Laws S., Lim F., Lim L., Tegg M., Lucas K., Martins G., Porter T., Rainey-Smith S., Rodrigues M., Shen K.K., Sohrabi H., Taddei K., Taddei T., Tan S., Verdile G., Weinborn M., Farrow M., Frost S., Hanson D., Hor M., Kanagasingam Y., Leifert W., Lockett L., Riley M., Saunders I., Thomas P. (2017). A blood-based biomarker panel indicates IL-10 and IL-12/23p40 are jointly associated as predictors of β-amyloid load in an AD cohort.. Sci. Rep..

[325] Eede P., Obst J., Benke E., Yvon-Durocher G., Richard B.C., Gimber N., Schmoranzer J., Böddrich A., Wanker E.E., Prokop S., Heppner F.L. (2020). Interleukin-/23 deficiency differentially affects pathology in male and female Alzheimer’s disease-like mice.. EMBO Rep..

[326] Porro C., Cianciulli A., Panaro M.A. (2020). The Regulatory Role of IL-10 in neurodegenerative diseases.. Biomolecules.

[327] Fei Z., Pan B., Pei R., Chen Z., Du X., Cao H., Li C. (2022). Efficacy and safety of blood derivatives therapy in Alzheimer’s disease: A systematic review and meta-analysis.. Syst. Rev..

[328] Rinne J.O., Brooks D.J., Rossor M.N., Fox N.C., Bullock R., Klunk W.E., Mathis C.A., Blennow K., Barakos J., Okello A.A. (2010). de LIano, S.R.M.; Liu, E.; Koller, M.; Gregg, K.M.; Schenk, D.; Black, R.; Grundman, M. 11C-PiB PET assessment of change in fibrillar amyloid-β load in patients with Alzheimer’s disease treated with bapineuzumab: A phase 2, double-blind, placebo-controlled, ascending-dose study.. Lancet Neurol..

[329] Vandenberghe R., Rinne J.O., Boada M., Katayama S., Scheltens P., Vellas B., Tuchman M., Gass A., Fiebach J.B., Hill D., Lobello K., Li D., McRae T., Lucas P., Evans I., Booth K., Luscan G., Wyman B.T., Hua L., Yang L., Brashear H.R., Black R.S. (2016). Bapineuzumab for mild to moderate Alzheimer’s disease in two global, randomized, phase 3 trials.. Alzheimers Res. Ther..

[330] Delnomdedieu M., Duvvuri S., Li D.J., Atassi N., Lu M., Brashear H.R., Liu E., Ness S., Kupiec J.W. (2016). First-In-Human safety and long-term exposure data for AAB-003 (PF-05236812) and biomarkers after intravenous infusions of escalating doses in patients with mild to moderate Alzheimer’s disease.. Alzheimers Res. Ther..

[331] Salloway S., Sperling R., Brashear H.R. (2014). Phase 3 trials of solanezumab and bapineuzumab for Alzheimer’s disease.. N. Engl. J. Med..

[332] Salloway S., Sperling R., Fox N.C., Blennow K., Klunk W., Raskind M., Sabbagh M., Honig L.S., Porsteinsson A.P., Ferris S., Reichert M., Ketter N., Nejadnik B., Guenzler V., Miloslavsky M., Wang D., Lu Y., Lull J., Tudor I.C., Liu E., Grundman M., Yuen E., Black R., Brashear H.R., Bapineuzumab 301 and 302 Clinical Trial Investigators (2014). Two phase 3 trials of bapineuzumab in mild-to-moderate Alzheimer’s disease.. N. Engl. J. Med..

[333] Honig L.S., Vellas B., Woodward M., Boada M., Bullock R., Borrie M., Hager K., Andreasen N., Scarpini E., Liu-Seifert H., Case M., Dean R.A., Hake A., Sundell K., Poole Hoffmann V., Carlson C., Khanna R., Mintun M., DeMattos R., Selzler K.J., Siemers E. (2018). Trial of solanezumab for mild dementia due to alzheimer’s disease.. N. Engl. J. Med..

[334] Salloway S., Farlow M., McDade E., Clifford D.B., Wang G., Llibre-Guerra J.J., Hitchcock J.M., Mills S.L., Santacruz A.M., Aschenbrenner A.J., Hassenstab J., Benzinger T.L.S., Gordon B.A., Fagan A.M., Coalier K.A., Cruchaga C., Goate A.A., Perrin R.J., Xiong C., Li Y., Morris J.C., Snider B.J., Mummery C., Surti G.M., Hannequin D., Wallon D., Berman S.B., Lah J.J., Jimenez-Velazquez I.Z., Roberson E.D., van Dyck C.H., Honig L.S., Sánchez-Valle R., Brooks W.S., Gauthier S., Galasko D.R., Masters C.L., Brosch J.R., Hsiung G.Y.R., Jayadev S., Formaglio M., Masellis M., Clarnette R., Pariente J., Dubois B., Pasquier F., Jack C.R., Koeppe R., Snyder P.J., Aisen P.S., Thomas R.G., Berry S.M., Wendelberger B.A., Andersen S.W., Holdridge K.C., Mintun M.A., Yaari R., Sims J.R., Baudler M., Delmar P., Doody R.S., Fontoura P., Giacobino C., Kerchner G.A., Bateman R.J., Formaglio M., Mills S.L., Pariente J., van Dyck C.H. (2021). A trial of gantenerumab or solanezumab in dominantly inherited Alzheimer’s disease.. Nat. Med..

[335] Geerts H., Walker M., Rose R., Bergeler S., van der Graaf P.H., Schuck E., Koyama A., Yasuda S., Hussein Z., Reyderman L., Swanson C., Cabal A. (2023). A combined physiologically-based pharmacokinetic and quantitative systems pharmacology model for modeling amyloid aggregation in Alzheimer’s disease.. CPT Pharmacometrics Syst. Pharmacol..

[336] Hettmann T., Gillies S.D., Kleinschmidt M., Piechotta A., Makioka K., Lemere C.A., Schilling S., Rahfeld J.U., Lues I. (2020). Development of the clinical candidate PBD-C06, a humanized pGlu3-Aβ-specific antibody against Alzheimer’s disease with reduced complement activation.. Sci. Rep..

[337] Mintun M.A., Lo A.C., Duggan Evans C., Wessels A.M., Ardayfio P.A., Andersen S.W., Shcherbinin S., Sparks J., Sims J.R., Brys M., Apostolova L.G., Salloway S.P., Skovronsky D.M. (2021). Donanemab in early Alzheimer’s disease.. N. Engl. J. Med..

[338] Lowe S.L., Duggan Evans C., Shcherbinin S., Cheng Y.J., Willis B.A., Gueorguieva I., Lo A.C., Fleisher A.S., Dage J.L., Ardayfio P., Aguiar G., Ishibai M., Takaichi G., Chua L., Mullins G., Sims J.R. (2021). Donanemab (LY3002813) Phase 1b study in alzheimer’s disease: Rapid and sustained reduction of brain amyloid measured by florbetapir F18 Imaging.. J. Prev. Alzheimers Dis..

[339] Gueorguieva I., Willis B.A., Chua L., Chow K., Ernest C.S., Shcherbinin S., Ardayfio P., Mullins G.R., Sims J.R. (2023). Donanemab population pharmacokinetics, amyloid plaque reduction, and safety in participants with Alzheimer’s disease.. Clin. Pharmacol. Ther..

[340] Sevigny J., Chiao P., Bussière T., Weinreb P.H., Williams L., Maier M., Dunstan R., Salloway S., Chen T., Ling Y., O’Gorman J., Qian F., Arastu M., Li M., Chollate S., Brennan M.S., Quintero-Monzon O., Scannevin R.H., Arnold H.M., Engber T., Rhodes K., Ferrero J., Hang Y., Mikulskis A., Grimm J., Hock C., Nitsch R.M., Sandrock A. (2016). The antibody aducanumab reduces Aβ plaques in Alzheimer’s disease.. Nature.

[341] Doroszkiewicz J., Mroczko B. (2022). New possibilities in the therapeutic approach to Alzheimer’s disease.. Int. J. Mol. Sci..

[342] Söderberg L., Johannesson M., Nygren P., Laudon H., Eriksson F., Osswald G., Möller C., Lannfelt L. (2023). Lecanemab, aducanumab, and gantenerumab: Binding profiles to different forms of amyloid-beta might explain efficacy and side effects in clinical trials for alzheimer’s disease.. Neurotherapeutics.

[343] Brandt N.J., Wheeler C., Courtin S.O. (2023). Navigating disease-modifying treatments for Alzheimer’s disease: Focusing on medications in phase 3 clinical trials.. J. Gerontol. Nurs..

[344] Yuksel J.M., Noviasky J., Britton S. (2022). Aducanumab for Alzheimer’s disease: Summarized data from emerge, engage, and prime studies.. Sr. Care Pharm..

[345] Swanson C.J., Zhang Y., Dhadda S., Wang J., Kaplow J., Lai R.Y.K., Lannfelt L., Bradley H., Rabe M., Koyama A., Reyderman L., Berry D.A., Berry S., Gordon R., Kramer L.D., Cummings J.L. (2021). A randomized, double-blind, phase 2b proof-of-concept clinical trial in early Alzheimer’s disease with lecanemab, an anti-Aβ protofibril antibody.. Alzheimers Res. Ther..

[346] Rafii M.S., Sperling R.A., Donohue M.C., Zhou J., Roberts C., Irizarry M.C., Dhadda S., Sethuraman G., Kramer L.D., Swanson C.J., Li D., Krause S., Rissman R.A., Walter S., Raman R., Johnson K.A., Aisen P.S. (2023). The AHEAD 3‐45 Study: Design of a prevention trial for Alzheimer’s disease.. Alzheimers Dement..

[347] Knopman D.S. (2023). Lecanemab reduces brain amyloid-β and delays cognitive worsening.. Cell Rep. Med..

[348] Piller C. (2023). Report on trial death stokes Alzheimer’s drug fears.. Science.

[349] Asuni A.A., Boutajangout A., Quartermain D., Sigurdsson E.M. (2007). Immunotherapy targeting pathological tau conformers in a tangle mouse model reduces brain pathology with associated functional improvements.. J. Neurosci..

[350] Boutajangout A., Ingadottir J., Davies P., Sigurdsson E.M. (2011). Passive immunization targeting pathological phospho-tau protein in a mouse model reduces functional decline and clears tau aggregates from the brain.. J. Neurochem..

[351] Yanamandra K., Patel T.K., Jiang H., Schindler S., Ulrich J.D., Boxer A.L., Miller B.L., Kerwin D.R., Gallardo G., Stewart F., Finn M.B., Cairns N.J., Verghese P.B., Fogelman I., West T., Braunstein J., Robinson G., Keyser J., Roh J., Knapik S.S., Hu Y., Holtzman D.M., Holtzman D.M. (2017). Anti-tau antibody administration increases plasma tau in transgenic mice and patients with tauopathy.. Sci. Transl. Med..

[352] Li L., Miao J., Jiang Y., Dai C.L., Iqbal K., Liu F., Chu D. (2023). Passive immunization inhibits tau phosphorylation and improves recognition learning and memory in 3xTg-AD mice.. Exp. Neurol..

[353] Novak P., Schmidt R., Kontsekova E., Zilka N., Kovacech B., Skrabana R., Vince-Kazmerova Z., Katina S., Fialova L., Prcina M., Parrak V., Dal-Bianco P., Brunner M., Staffen W., Rainer M., Ondrus M., Ropele S., Smisek M., Sivak R., Winblad B., Novak M. (2017). Safety and immunogenicity of the tau vaccine AADvac1 in patients with Alzheimer’s disease: A randomised, double-blind, placebo-controlled, phase 1 trial.. Lancet Neurol..

[354] Novak P., Zilka N., Zilkova M., Kovacech B., Skrabana R., Ondrus M., Fialova L., Kontsekova E., Otto M., Novak M. (2019). AADvac1, an active immunotherapy for Alzheimer’s disease and non alzheimer tauopathies: An overview of preclinical and clinical development.. J. Prev. Alzheimers Dis..

[355] Hovakimyan A., Zagorski K., Chailyan G., Antonyan T., Melikyan L., Petrushina I., Batt D.G., King O., Ghazaryan M., Donthi A., Foose C., Petrovsky N., Cribbs D.H., Agadjanyan M.G., Ghochikyan A. (2022). Immunogenicity of MultiTEP platform technology-based Tau vaccine in non-human primates.. NPJ Vaccines.

[356] Pagano G., Boess F.G., Taylor K.I., Ricci B., Mollenhauer B., Poewe W., Boulay A., Anzures-Cabrera J., Vogt A., Marchesi M., Post A., Nikolcheva T., Kinney G.G., Zago W.M., Ness D.K., Svoboda H., Britschgi M., Ostrowitzki S., Simuni T., Marek K., Koller M., Sevigny J., Doody R., Fontoura P., Umbricht D., Bonni A. (2021). A Phase II study to evaluate the safety and efficacy of prasinezumab in early parkinson’s disease (PASADENA): Rationale, design, and baseline data.. Front. Neurol..

[357] Pagano G., Taylor K.I., Anzures-Cabrera J., Marchesi M., Simuni T., Marek K., Postuma R.B., Pavese N., Stocchi F., Azulay J.P., Mollenhauer B., López-Manzanares L., Russell D.S., Boyd J.T., Nicholas A.P., Luquin M.R., Hauser R.A., Gasser T., Poewe W., Ricci B., Boulay A., Vogt A., Boess F.G., Dukart J., D’Urso G., Finch R., Zanigni S., Monnet A., Pross N., Hahn A., Svoboda H., Britschgi M., Lipsmeier F., Volkova-Volkmar E., Lindemann M., Dziadek S., Holiga Š., Rukina D., Kustermann T., Kerchner G.A., Fontoura P., Umbricht D., Doody R., Nikolcheva T., Bonni A. (2022). Trial of prasinezumab in early-stage parkinson’s disease.. N. Engl. J. Med..

[358] Kuchimanchi M., Monine M., Kandadi M.K., Woodward C., Penner N., Phase I.I. (2020). Phase II dose selection for alpha synuclein–targeting antibody cinpanemab (BIIB054) based on target protein binding levels in the brain.. CPT Pharmacometrics Syst. Pharmacol..

[359] Lang A.E., Siderowf A.D., Macklin E.A., Poewe W., Brooks D.J., Fernandez H.H., Rascol O., Giladi N., Stocchi F., Tanner C.M., Postuma R.B., Simon D.K., Tolosa E., Mollenhauer B., Cedarbaum J.M., Fraser K., Xiao J., Evans K.C., Graham D.L., Sapir I., Inra J., Hutchison R.M., Yang M., Fox T., Budd Haeberlein S., Dam T. (2022). Trial of cinpanemab in early parkinson’s disease.. N. Engl. J. Med..

[360] Schofield D.J., Irving L., Calo L., Bogstedt A., Rees G., Nuccitelli A., Narwal R., Petrone M., Roberts J., Brown L., Cusdin F., Dosanjh B., Lloyd C., Dobson C., Gurrell I., Fraser G., McFarlane M., Rockenstein E., Spencer B., Masliah E., Spillantini M.G., Tan K., Billinton A., Vaughan T., Chessell I., Perkinton M.S., Perkinton M.S. (2019). Preclinical development of a high affinity α-synuclein antibody, MEDI1341, that can enter the brain, sequester extracellular α-synuclein and attenuate α-synuclein spreading in vivo.. Neurobiol. Dis..

[361] Fjord-Larsen L., Thougaard A., Wegener K.M., Christiansen J., Larsen F., Schrøder-Hansen L.M., Kaarde M., Ditlevsen D.K. (2021). Nonclinical safety evaluation, pharmacokinetics, and target engagement of Lu AF82422, a monoclonal IgG1 antibody against alpha-synuclein in development for treatment of synucleinopathies.. MAbs.

[362] Kallab M., Herrera-Vaquero M., Johannesson M., Eriksson F., Sigvardson J., Poewe W., Wenning G.K., Nordström E., Stefanova N. (2018). Region-specific effects of immunotherapy with antibodies targeting α-synuclein in a transgenic model of synucleinopathy.. Front. Neurosci..

[363] Nordström E., Eriksson F., Sigvardson J., Johannesson M., Kasrayan A., Jones-Kostalla M., Appelkvist P., Söderberg L., Nygren P., Blom M., Rachalski A., Nordenankar K., Zachrisson O., Amandius E., Osswald G., Moge M., Ingelsson M., Bergström J., Lannfelt L., Möller C., Giorgetti M., Fälting J. (2021). ABBV-0805, a novel antibody selective for soluble aggregated α-synuclein, prolongs lifespan and prevents buildup of α-synuclein pathology in mouse models of Parkinson’s disease.. Neurobiol. Dis..

[364] Gibbs E., Zhao B., Roman A., Plotkin S.S., Peng X., Hsueh S.C.C., Aina A., Wang J., Shyu C., Yip C.K., Nam S.E., Kaplan J.M., Cashman N.R. (2022). Rational generation of monoclonal antibodies selective for pathogenic forms of alpha-synuclein.. Biomedicines.

[365] Valiukas Z., Ephraim R., Tangalakis K., Davidson M., Apostolopoulos V., Feehan J. (2022). Immunotherapies for Alzheimer’s disease: A review.. Vaccines.

[366] Knecht L., Folke J., Dodel R., Ross J.A., Albus A. (2022). Alpha-synuclein immunization strategies for synucleinopathies in clinical studies: A biological perspective.. Neurotherapeutics.

[367] Meissner W.G., Traon A.P.L., Foubert-Samier A., Galabova G., Galitzky M., Kutzelnigg A., Laurens B., Lührs P., Medori R., Péran P., Sabatini U., Vergnet S., Volc D., Poewe W., Schneeberger A., Staffler G., Rascol O., Anheim M., Castrioto A., Derkinderen P., Drapier S., Eusebio A., Grabli D., Marques A., Moreau C., Moro E., Tranchant C. (2020). A Phase 1 Randomized Trial of Specific Active α-SYNUCLEIN Immunotherapies PD01A and PD03A in Multiple System Atrophy.. Mov. Disord..

[368] Yu H.J., Thijssen E., van Brummelen E., van der Plas J.L., Radanovic I., Moerland M., Hsieh E., Groeneveld G.J., Dodart J.C. (2022). A randomized first-in-human study with UB-312, a UBITh® α-synuclein peptide vaccine.. Mov. Disord..

[369] Nimmo J.T., Smith H., Wang C.Y., Teeling J.L., Nicoll J.A.R., Verma A., Dodart J-C., Liu Z., Lin F., Carare R.O. (2022). Immunisation with UB-312 in the Thy1SNCA mouse prevents motor performance deficits and oligomeric α-synuclein accumulation in the brain and gut.. Acta Neuropathol..

[370] Schmidhuber S., Scheiblhofer S., Weiss R., Cserepes M., Tóvári J., Gadermaier G., Bezard E., De Giorgi F., Ichas F., Strunk D., Mandler M. (2022). A Novel C-type lectin receptor-targeted α-synuclein-based parkinson vaccine induces potent immune responses and therapeutic efficacy in mice.. Vaccines.

[371] Chen Z., Yang Y., Yang X., Zhou C., Li F., Lei P., Zhong L., Jin X., Peng G. (2013). Immune effects of optimized DNA vaccine and protective effects in a MPTP model of Parkinson’s disease.. Neurol. Sci..

[372] Petrushina I., Hovakimyan A., Harahap-Carrillo I.S., Davtyan H., Antonyan T., Chailyan G., Kazarian K., Antonenko M., Jullienne A., Hamer M.M., Obenaus A., King O., Zagorski K., Blurton-Jones M., Cribbs D.H., Lander H., Ghochikyan A., Agadjanyan M.G. (2020). Characterization and preclinical evaluation of the cGMP grade DNA based vaccine, AV-1959D to enter the first-in-human clinical trials.. Neurobiol. Dis..

[373] Kim C., Hovakimyan A., Zagorski K., Antonyan T., Petrushina I., Davtyan H., Chailyan G., Hasselmann J., Iba M., Adame A., Rockenstein E., Szabo M., Blurton-Jones M., Cribbs D.H., Ghochikyan A., Masliah E., Agadjanyan M.G. (2022). Efficacy and immunogenicity of MultiTEP-based DNA vaccines targeting human α-synuclein: Prelude for IND enabling studies.. NPJ Vaccines.

[374] Masliah E., Rockenstein E., Mante M., Crews L., Spencer B., Adame A., Patrick C., Trejo M., Ubhi K., Rohn T.T., Mueller-Steiner S., Seubert P., Barbour R., McConlogue L., Buttini M., Games D., Schenk D. (2011). Passive immunization reduces behavioral and neuropathological deficits in an alpha-synuclein transgenic model of Lewy body disease.. PLoS One.

[375] Nimmo J.T., Verma A., Dodart J.C., Wang C.Y., Savistchenko J., Melki R., Carare R.O., Nicoll J.A.R. (2020). Novel antibodies detect additional α-synuclein pathology in synucleinopathies: Potential development for immunotherapy.. Alzheimers Res. Ther..

[376] Games D., Valera E., Spencer B., Rockenstein E., Mante M., Adame A., Patrick C., Ubhi K., Nuber S., Sacayon P., Zago W., Seubert P., Barbour R., Schenk D., Masliah E. (2014). Reducing C-terminal-truncated alpha-synuclein by immunotherapy attenuates neurodegeneration and propagation in Parkinson’s disease-like models.. J. Neurosci..

[377] Wang S., Yu Y., Geng S., Wang D., Zhang L., Xie X., Wu B., Li C., Xu H., Li X., Hu Y., Zhang L., Kaether C., Wang B. (2014). A coimmunization vaccine of Aβ42 ameliorates cognitive deficits without brain inflammation in an Alzheimer’s disease model.. Alzheimers Res. Ther..

[378] Xiao B., Tan E.K. (2023). Immunotherapy trials in parkinson’s disease: Challenges.. J. Transl. Med..

[379] Nicoll J.A.R., Buckland G.R., Harrison C.H., Page A., Harris S., Love S., Neal J.W., Holmes C., Boche D. (2019). Persistent neuropathological effects 14 years following amyloid-β immunization in Alzheimer’s disease.. Brain.

[380] Chu W.T., Hall J., Gurrala A., Becsey A., Raman S., Okun M.S., Flores C.T., Giasson B.I., Vaillancourt D.E., Vedam-Mai V. (2023). Evaluation of an adoptive cellular therapy-based vaccine in a transgenic mouse model of α-synucleinopathy.. ACS Chem. Neurosci..

[381] Olson K.E., Namminga K.L., Schwab A.D., Thurston M.J., Lu Y., Woods A., Lei L., Shen W., Wang F., Joseph S.B., Gendelman H.E., Mosley R.L. (2020). Neuroprotective activities of long-acting granulocyte–macrophage colony-stimulating factor (mpdm608) in 1-methyl-4-phenyl-1,2,3,6-tetrahydropyridine-intoxicated mice.. Neurotherapeutics.

[382] Olson K.E., Namminga K.L., Lu Y., Schwab A.D., Thurston M.J., Abdelmoaty M.M., Kumar V., Wojtkiewicz M., Obaro H., Santamaria P., Mosley R.L., Gendelman H.E. (2021). Safety, tolerability, and immune-biomarker profiling for year-long sargramostim treatment of Parkinson’s disease.. EBioMedicine.

[383] Gendelman H.E., Zhang Y., Santamaria P., Olson K.E., Schutt C.R., Bhatti D., Shetty B.L.D., Lu Y., Estes K.A., Standaert D.G., Heinrichs-Graham E., Larson L., Meza J.L., Follett M., Forsberg E., Siuzdak G., Wilson T.W., Peterson C., Mosley R.L. (2017). Evaluation of the safety and immunomodulatory effects of sargramostim in a randomized, double-blind phase 1 clinical Parkinson’s disease trial.. NPJ Parkinsons Dis..

[384] Rohrer L., Yunce M., Montine T.J., Shan H. (2023). Plasma exchange in Alzheimer’s disease.. Transfus. Med. Rev..

[385] Boada M., López O.L., Olazarán J., Núñez L., Pfeffer M., Paricio M., Lorites J., Piñol-Ripoll G., Gámez J.E., Anaya F., Kiprov D., Lima J., Grifols C., Torres M., Costa M., Bozzo J., Szczepiorkowski Z.M., Hendrix S., Páez A. (2020). A randomized, controlled clinical trial of plasma exchange with albumin replacement for Alzheimer’s disease: Primary results of the AMBAR Study.. Alzheimers Dement..

[386] Singh S., Kumar K., Panda M., Srivastava A., Mishra A., Prajapati V.K. (2023). High-throughput virtual screening of small-molecule inhibitors targeting immune cell checkpoints to discover new immunotherapeutics for human diseases.. Mol. Divers..

[387] Liu Y., Meng Y., Zhou C., Yan J., Guo C., Dong W. (2023). Activation of the IL-17/TRAF6/NF-κB pathway is implicated in Aβ-induced neurotoxicity.. BMC Neurosci..

[388] Badr M., McFleder R.L., Wu J., Knorr S., Koprich J.B., Hünig T., Brotchie J.M., Volkmann J., Lutz M.B., Ip C.W. (2022). Expansion of regulatory T cells by CD28 superagonistic antibodies attenuates neurodegeneration in A53T-α-synuclein Parkinson’s disease mice.. J. Neuroinflammation.

